# Limited overlap in significant hits between genome-wide association studies on two airflow obstruction definitions in the same population

**DOI:** 10.1186/s12890-019-0811-0

**Published:** 2019-03-07

**Authors:** Diana A. van der Plaat, Judith M. Vonk, Lies Lahousse, Kim de Jong, Alen Faiz, Ivana Nedeljkovic, Najaf Amin, Cleo C. van Diemen, Guy G. Brusselle, Yohan Bossé, Corry-Anke Brandsma, Ke Hao, Peter D. Paré, Cornelia M. van Duijn, Dirkje S. Postma, H. Marike Boezen

**Affiliations:** 1 Department of Epidemiology, University of Groningen, University Medical Center Groningen, Hanzeplein 1, 9700 RB Groningen, The Netherlands; 2 Groningen Research Institute for Asthma and COPD (GRIAC), University of Groningen, University Medical Center Groningen, Groningen, The Netherlands; 3 Department of Epidemiology, Erasmus Medical Center, Rotterdam, The Netherlands; 4 Department of Respiratory Medicine, Ghent University Hospital, Ghent, Belgium; 5 Department of Pathology and Medical Biology, University of Groningen, University Medical Center Groningen, Groningen, The Netherlands; 6 Department of Genetics, University of Groningen, University Medical Center Groningen, Groningen, The Netherlands; 7 Department of Respiratory Medicine, Erasmus Medical Center, Rotterdam, The Netherlands; 8 Department of Molecular Medicine, Institut universitaire de cardiologie et de pneumologie de Québec, Laval University, Québec, Canada; 9 Merck Research Laboratories, Boston, MA USA; 10 Department of Medicine, Center for Heart Lung Innovation and Institute for Heart and Lung Health, University of British Columbia, St. Paul’s Hospital, Vancouver, Canada; 11 Department of Pulmonary Diseases, University of Groningen, University Medical Center Groningen, Groningen, The Netherlands

**Keywords:** Genome-wide association study, Genetics, Airflow obstruction, COPD

## Abstract

**Background:**

Airflow obstruction is a hallmark of chronic obstructive pulmonary disease (COPD), and is defined as either the ratio between forced expiratory volume in one second and forced vital capacity (FEV_1_/FVC) < 70% or < lower limit of normal (LLN). This study aimed to assess the overlap between genome-wide association studies (GWAS) on airflow obstruction using these two definitions in the same population stratified by smoking.

**Methods:**

GWASes were performed in the LifeLines Cohort Study for both airflow obstruction definitions in never-smokers (NS = 5071) and ever-smokers (ES = 4855). The FEV_1_/FVC < 70% models were adjusted for sex, age, and height; FEV_1_/FVC < LLN models were not adjusted. Ever-smokers models were additionally adjusted for pack-years and current-smoking. The overlap in significantly associated SNPs between the two definitions and never/ever-smokers was assessed using several *p*-value thresholds. To quantify the agreement, the Pearson correlation coefficient was calculated between the *p*-values and ORs. Replication was performed in the Vlagtwedde-Vlaardingen study (NS = 432, ES = 823). The overlapping SNPs with *p* < 10^− 4^ were validated in the Vlagtwedde-Vlaardingen and Rotterdam Study cohorts (NS = 1966, ES = 3134) and analysed for expression quantitative trait loci (eQTL) in lung tissue (*n* = 1087).

**Results:**

In the LifeLines cohort, 96% and 93% of the never- and ever-smokers were classified concordantly based on the two definitions. 26 and 29% of the investigated SNPs were overlapping at *p* < 0.05 in never- and ever-smokers, respectively. At p < 10^− 4^ the overlap was 4% and 6% respectively, which could be change findings as shown by simulation studies. The effect estimates of the SNPs of the two definitions correlated strongly, but the p-values showed more variation and correlated only moderately. Similar observations were made in the Vlagtwedde-Vlaardingen study. Two overlapping SNPs in never-smokers (*NFYC* and *FABP7)* had the same direction of effect in the validation cohorts and the *NFYC* SNP was an eQTL for *NFYC-AS1*. *NFYC* is a transcription factor that binds to several known COPD genes, and *FABP7* may be involved in abnormal pulmonary development.

**Conclusions:**

The definition of airflow obstruction and the population under study may be important determinants of which SNPs are associated with airflow obstruction. The genes *FABP7* and *NFYC(-AS1)* could play a role in airflow obstruction in never-smokers specifically.

**Electronic supplementary material:**

The online version of this article (10.1186/s12890-019-0811-0) contains supplementary material, which is available to authorized users.

## Background

Chronic obstructive pulmonary disease (COPD) is a major cause of morbidity and mortality in the world and encompasses emphysema, chronic bronchitis, and small airways disease [1, 2]. The diagnosis of COPD is largely based on the presence of airflow obstruction, measured by the spirometric assessment (post-bronchodilator) of the ratio between forced expiratory volume in one second and forced vital capacity (FEV_1_/FVC). The Global initiative for chronic Obstructive Lung Disease (GOLD) recommends to use a fixed cut-off for defining airflow obstruction, namely an FEV_1_/FVC ratio below 70% [3], whereas the American Thoracic Society/European Respiratory Society (ATS/ERS) guidelines recommend to define airflow obstruction as FEV_1_/FVC below the lower limit of normal (LLN) [4]. The LLN is a reference value based on sex, age, height and ethnicity and is calculated as the lower fifth percentile of a healthy reference population [5]. There is a considerable controversy about which definition should be used in research and clinical practice, since both may lead to misclassifications [5–8]. This has important implications, since misclassifications may lead to inappropriate medication and therapies [9, 10].

It is generally accepted that both genetic susceptibility and environmental factors contribute to airflow obstruction. Genetic variants associated with airflow obstruction have been identified by several genome-wide association studies (GWAS), but different definitions of airflow obstruction and populations were used. [11–16] As an illustration, the case-control study including only smokers with > 2.5 pack-years by Pillai et al. used the fixed ratio (FEV_1_/FVC < 70%) to define airflow obstruction, while the population based study including both ever- and never-smokers by Wilk et al. used the lower limit of normal (LLN) [15, 16]. Only few regions were identified in both studies, namely the *CHRNA5/3* and *HHIP* regions. We therefore aimed to assess the genetic overlap between the two definitions of airflow obstruction in the same individuals. We stratified by smoking status to assess the overlap between the two airflow obstruction definitions in never- and ever-smokers separately. We used the Lifelines Cohort Study as discovery sample and the Vlagtwedde-Vlaardingen study to replicate our observations. In addition, genetic loci associated with both airflow obstruction definitions could indicate robust genetic associations with airflow obstruction, which could potentially be novel loci. We therefore, as a secondary aim, validated the top overlapping single-nucleotide polymorphisms (SNPs) between the two airflow obstruction discovery analyses in an independent SNP validation sample and assessed if they were acting as expression quantitative trait loci (eQTLs) in a lung tissue sample.

## Materials and methods

### Study populations

To study the overlap between the two airflow obstruction definitions, all subjects with available genotypic data were included from the Dutch LifeLines Cohort Study (discovery sample) and the Vlagtwedde-Vlaardingen study (replication sample) [17–19]. In addition, subjects from the Vlagtwedde-Vlaardingen study and the three independent cohorts of the Rotterdam Study (RS I to III) were selected to validate the top overlapping SNPs from LifeLines (SNP validation sample), thereby increasing the SNP validation sample size [20]. All subjects provided written informed consent and the studies were approved by local medical ethics committees. Smoking status was based on self-reported smoking history and pack-years smoked. In the stratified analyses never-smokers having smoked 0 pack-years and ever-smokers having smoked > 5 pack-years were included, thereby excluding subjects with > 0 and ≤ 5 pack-years. Subjects were defined as having airflow obstruction based on having a pre-bronchodilator FEV_1_/FVC ratio (%) < 70% or < LLN (based on Global Lung Initiative 2012 (GLI-2012)) [21]. All subjects completed pulmonary function testing according to ATS or ERS criteria [22]. Additional details are provided in Additional file 1.

### Genotyping

The IlluminaCytoSNP-12 arrays were used to genotype blood samples in LifeLines and the Vlagtwedde-Vlaardingen study. SNPs with a genotype call-rate ≥ 95%, minor allele frequency ≥ 1% and Hardy-Weinberg *p*-value ≥10^− 4^ were included. Non-Caucasian samples and first-degree relatives were excluded based on self-reporting, outlier (Identity By State) and principal component analysis. After quality control, 227,981 genotyped SNPs were included in the discovery analyses (LifeLines) and 242,926 genotyped SNPs were included in the replication analyses (Vlagtwedde-Vlaardingen). Only genotyped SNPs were included in the analyses to prevent introducing bias, since it is known that imputation can reduce the effect size estimation, especially if healthy controls are used as reference [23]. Blood samples in the Rotterdam study were genotyped with the 610 K and 660 K Illumina arrays and similar QC criteria as in the other cohorts were applied.

### Statistical analysis

Four separate GWASes were performed assessing the genetic associations between the two definitions of airflow obstruction, stratified by smoking status, for both the LifeLines (discovery) and Vlagtwedde-Vlaardingen (replication) studies. Logistic regression (additive genetic model) was performed using PLINK (v1.07) [24]. The “FEV_1_/FVC < 70%” model was adjusted for sex, age and height. The “FEV_1_/FVC < LLN” model was not adjusted for these variables, since they are included in the LLN calculation. In ever-smokers, the models were additionally adjusted for pack-years and current-smoking. We used different *p*-value thresholds to assess the number of overlapping SNPs between the two definitions. In addition, to quantify the agreement of the results between the two definitions and between never-and ever-smokers, we calculated the Pearson correlation coefficient between the *p*-values and between the ORs.

### Power simulations

Or study has a relative small sample size (*n* = 5070) and therefore relative low power. We assessed the effect of low power on the overlap between the two definitions by increasing our never-smoking discovery sample (LifeLines) 2 (*n* = 10,140) and 4 (*n* = 20,280) times. In addition, to assess if our results were spurious, we used our never-smoking discovery sample and randomly allocated 10 times the airflow obstruction cases but keeping the same distribution as in our original dataset (FEV_1_/FVC < 70%: *n* = 548, FEV_1_/FVC < LLN: *n* = 401, overlapping cases: *n* = 371 (64%)). For both simulation studies, we repeated the GWAS analyses on both airflow obstruction definitions in the created datasets and compared the number of overlapping SNPs.

### Validation of overlapping SNPs

Only the top overlapping SNPs between the two airflow obstruction definitions in the discovery sample (LifeLines) were evaluated in the SNP validation sample, the Vlagtwedde-Vlaardingen study and RS I to III. A fixed-effects meta-analysis of the effect estimates weighted by the inverse of the standard errors from all four validation cohorts was performed using METAL (v2011) [25]. We considered replication if the meta-analysis *p*-value was below the Bonferroni corrected p-value defined as 0.05/number of overlapping SNPs and, in addition, had the same direction of effect in all cohorts. In addition, SNP*ever-smoking interactions were estimated and we assessed if the overlapping SNPs were associated with gene expression levels in lung tissue within a 4 Mb window around the SNP (2 Mb on either side of the SNP), using data from the lung eQTL consortium [26]. In total, 1087 subjects were included in the linear regression model, adjusted for disease status, age, sex, smoking, and cohort specific principal components. SNPs with a p-value below the Bonferroni corrected threshold (*p* = 0.05/number of probesets) were considered significant eQTLs. See Additional file 1 and GEO accession numbers GSE23546 and GPL10379 for additional information.

## Results

### Population characteristics

**Fig. 1:**
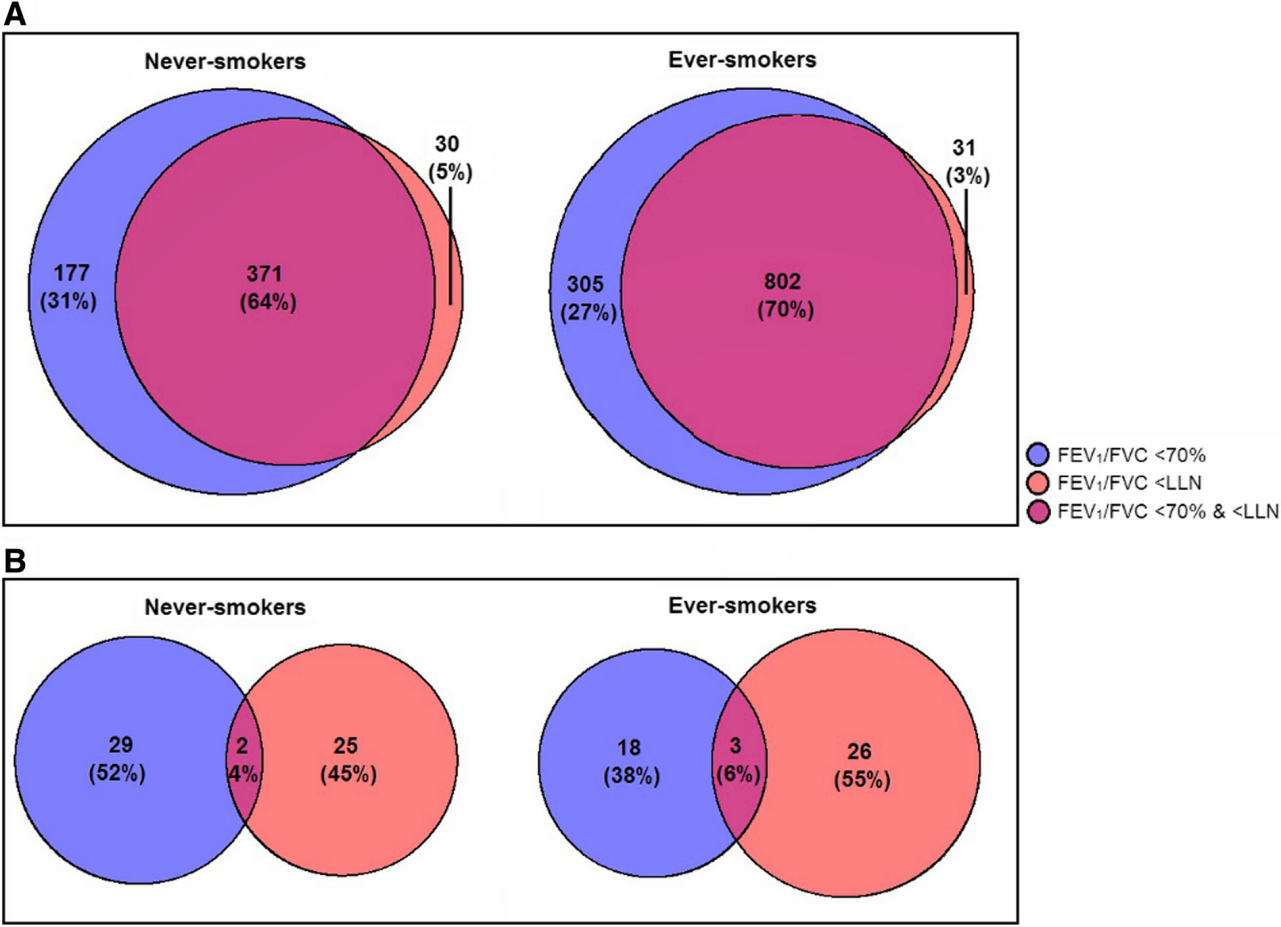
Venn diagrams showing the overlap between the two definitions of airflow obstruction for the number of subjects classified as having airflow obstruction (**a**) and the number of identified SNPs with *p* < 10^− 4^ (**b**) in LifeLines (discovery sample)

The LifeLines cohort (discovery sample) included 5070 never-smokers and 4855 ever-smokers with complete data on all covariates (see Table 1). Of the never-smokers in LifeLines, 96% had a concordant airflow obstruction classification for the two definitions: 89% did not have airflow obstruction and 7% did have airflow obstruction. The remaining 4% had a discordant classification (see Additional file 1: Table S1A). Figure 1a shows that of all never-smoking subjects with airflow obstruction based on at least one airflow obstruction definition (*n* = 578), 36% had a discordant airflow obstruction classification. Of the ever-smokers, 93% was classified concordantly: 77% did not have airflow obstruction and 17% did have airflow obstruction. The remaining 7% had a discordant classification (see Additional file 1: Table S1B). Of all ever-smoking subjects with airflow obstruction based on at least one definition (*n* = 1138), 30% had a discordant airflow obstruction classification (see Fig. 1a). Subjects with an FEV_1_/FVC < 70% and > LLN were aged between 41 and 85, and subjects with an FEV_1_/FVC > 70% and < LLN were aged between 22 and 43. These and other characteristics of the airflow obstruction groups separately for never- and ever-smokers in LifeLines are shown in Additional file 1: Table S2.

**Table 1:** Characteristics of never- and ever-smokers included in the current study

	Never-smokers					Ever-smokers				
	LifeLines	Vla-Vla^b^	RS I	RS II	RS III	LifeLines	Vla-Vla^b^	RS I	RS II	RS III
N with no missing data	5070	432	408	379	747	4855	823	640	583	1088
Males, N (%)	1942 (38)	103 (23)	81 (20)	124 (33)	308 (41)	2312 (48)	590 (72)	375 (59)	343 (59)	531 (49)
Age (yrs), median (min-max)	46 (18–89)	54 (36–79)	78 (72–94)	71 (65–98)	62 (51–93)	49 (22–85)	53 (35–79)	79 (72–95)	71 (65–93)	62 (52–93)
Height (cm), mean (SD)	174 (9)	166 (9)	163 (9)	166 (9)	171 (9)	175 (9)	173 (8)	168 (9)	171 (9)	172 (9)
Current-smokers, N (%)	–	–	–	–	–	2171 (45)	478 (58)	99 (16)	108 (19)	298 (27)
Pack-years (yrs), mean (SD)	–	–	–	–	–	17 (11)	27 (21)	29 (21)	29 (21)	27 (20)
Pulmonary function, mean (SD)										
FEV_1_%predicted (%), mean (SD)^a^	98 (13)	91 (13)	101 (18)	102 (16)	101 (16)	94 (14)	85 (15)	98 (23)	96 (21)	99 (19)
FEV_1_/FVC (%), mean (SD)^b^	78 (7)	76 (7)	77 (7)	79 (6)	78 (6)	75 (8)	72 (10)	74 (9)	74 (9)	75 (8)
FEV_1_/FVC <70%, N (%)	548 (11)	77 (18)	51 (13)	27 (7)	75 (10)	1107 (23)	287 (35)	163 (26)	151 (26)	235 (22)
FEV_1_/FVC < LLN, N (%)	401 (8)	49 (11)	9 (2)	9 (2)	25 (3)	833 (17)	212 (26)	63 (10)	62 (11)	122 (11)
Both FEV_1_/FVC <70% and < LLN, N (%)	371 (7)	49 (11)	9 (2)	9 (2)	25 (3)	802 (17)	207 (25)	63 (10)	62 (11)	122 (11)
Moderate/severe COPD, N (%)^*c*^	106 (2)	26 (6)	21 (5)	9 (2)	16 (2)	310 (6)	145 (18)	90 (14)	90 (15)	104 (10)

Discovery sample = LifeLines cohort study, replication samples = Vlagtwedde-Vlaardingen (Vla-Vla) study, and SNP validation sample = Vla-Vla and RS I to III

*FEV*_*1*_ forced expiratory volume in one second, *FVC* forced vital capacity

^a^FEV_1_%predicted is based on the reference equation by GLI-2012 [21]

^b^FEV_1_%IVC (inspiratory vital capacity) for the Vlagtwedde-Vlaardingen study (Vla-Vla)

^c^COPD GOLD stage 2 and up (FEV_1_/FVC <70% and FEV_1_%*p* < 80% based on pre-bronchodilator measurements)

The Vlagtwedde-Vlaardingen study (replication sample) included 432 never-smokers and 823 ever-smokers (see Table 1). Of the Vlagtwedde-Vlaardingen study, 94% and 90% of the never- and ever-smokers were classified concordantly based on the two definitions (see Additional file 1: Table S1 C-D).

The SNP validation sample used for the SNP validation meta-analysis included 1966 never-smokers and 3134 ever-smokers from the Vlagtwedde-Vlaardingen study and RS I to III (see Table 1).

### GWAS results

There was minimal population stratification in all analyses of LifeLines, indicated by the genomic inflation factor lambda (λ: 1.0002–1.0217, see Additional file 1: Figure S1). The results based on a *p* < 10^− 4^ of all four analyses in LifeLines are given in See (Additional file 1: Tables S3-S6), including the Manhattan plots (see Additional file 1: Figures S2 and S3). For comparison, the effect estimates of both airflow obstruction definition are given in these tables. Summary statistics (*p* values, betas, and standard errors for all SNPs that were tested) of the GWAS result of both the Lifelines Cohort Study and the Vlagtwedde-Vlaardingen study are provided in Additional file 2.

### Overlap between the results

We used several *p*-value thresholds to assess the overlap between the GWAS results of both airflow obstruction definitions separately in never- and ever-smokers of LifeLines (see Table 2). A threshold of 0.05 resulted in the observation that 26% and 29% of the SNPs were overlapping between the two airflow obstruction definitions in never- and ever-smokers, respectively. Three percent of the SNPs were overlapping between never- and ever-smokers for both definitions. A smaller *p*-value threshold resulted in a lower percentage of overlap e.g. a threshold of p < 10^− 4^ resulted in 4% and 6% overlapping SNPs between the two airflow obstruction definitions in never- and ever-smokers, respectively (see Fig. 1b), and zero overlap between never- and ever-smokers using the same definition of airflow obstruction. Similar observations were made in the replication sample the Vlagtwedde-Vlaardingen study (see Table 2), since at *p* < 0.05 the overlap between the definitions was 24% and 25% in never- and ever-smokers, respectively, and 2% of the SNPs were overlapping between never- and ever-smokers for both definitions.

**Table 2:** Table showing the number of SNPs with a *p*-value below the mentioned threshold for both FEV_1_/FVC < 70% and < LLN analysis and the overlap

Threshold	Never-smokers			Ever-smokers (> 5 py)			Overlap never- and ever-smokers	
	70%	LLN	Overlap	70%	LLN	Overlap	70%	LLN
*Discovery analysis (LifeLines)*								
< 0.05	11,377	11,475	4755 (26%)	12,114	12,366	5445 (29%)	625 (2.7%)	616 (2.7%)
<0.01	2232	2197	673 (18%)	2522	2493	824 (20%)	23 (0.5%)	21 (0.4%)
< 10^−3^	222	233	54 (13%)	246	266	76 (17%)	1 (0.2%)	0
< 10^− 4^	31	27	2 (4%)	21	29	3 (6%)	0	0
< 10^−5^	4	4	0	3	2	1 (25%)	0	0
< 10^− 6^	1	0	0	0	0	0	0	0
<Bonferroni*	0	0	0	0	0	0	0	0
*Replication analysis (Vlagtwedde-Vlaardingen study)*								
<0.05	10,702	10,295	4026 (24%)	12,592	12,571	4976 (25%)	487 (2.1%)	488 (2.2%)
<0.01	1857	1807	544 (17%)	2567	2609	754 (17%)	17 (0.4%)	12 (0.3%)
< 10^−3^	164	174	38 (13%)	223	262	41 (9%)	0	0
< 10^−4^	18	19	6 (19%)	12	21	0	0	0
< 10^−5^	7	3	2 (25%)	0	0	0	0	0
< 10^−6^	1	0	0	0	0	0	0	0
<Bonferroni*	0	0	0	0	0	0	0	0

*P < 2.19 × 10^−7^

**Fig. 2:**
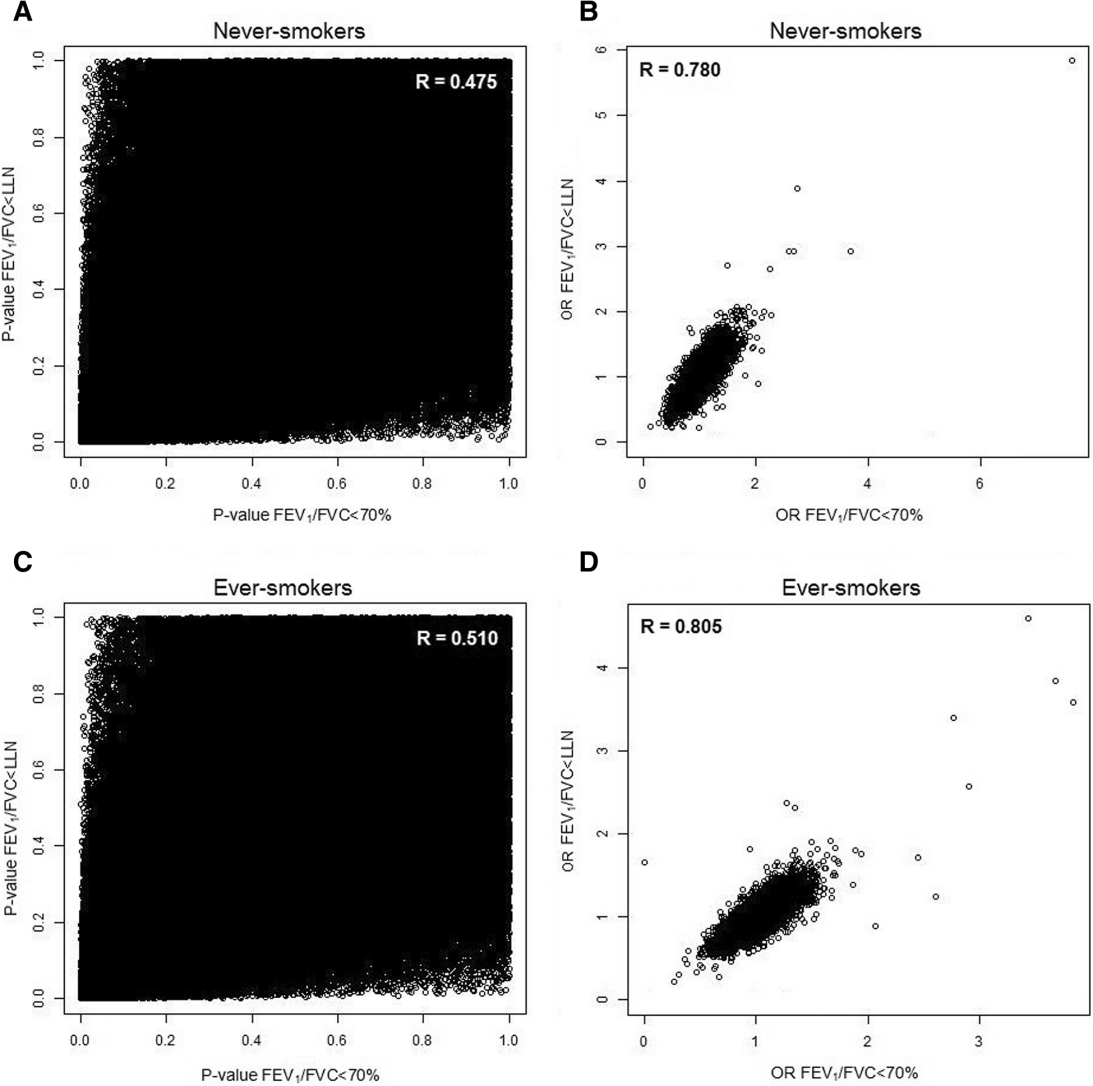
Pearson correlation between the p-values (**a**/**c**) or OR (B/D) of FEV_1_/FVC < 70% and < LLN analyses separately for never- (**a**/**b**) and ever-smokers (**c**/**d**) in LifeLines (discovery sample)

The correlations between the SNP-specific *p*-values and ORs from the two airflow obstruction definitions were 0.48 (*p*-value) and 0.78 (OR) in never-smokers, and 0.51 (*p*-value) and 0.81 (OR) in ever-smokers (see Fig. 2). Between never- and ever-smokers the correlations of the SNP-specific p-values were 0.0008 for FEV_1_/FVC < 70% and 0.002 for FEV_1_/FVC < LLN, and for the OR the correlation was − 0.02 for both definitions. Similar observations were made in the replication sample, the Vlagtwedde-Vlaardingen study. The correlations between the two definitions were 0.45 (*p*-value) and 0.76 (OR) in never-smokers, and 0.41 (*p*-value) and 0.74 (OR) in ever-smokers. Between never- and ever-smokers the correlations were − 0.001 (*p*-value) and 0.015 (OR) for FEV_1_/FVC < 70% and − 0.003 (*p*-value) and 0.004 (OR) for FEV_1_/FVC < LLN.

### Power simulations

We found that the percentage of overlap increased when we expanded our never-smoking identification sample 2 and 4 times (see Additional file 1: Table S7). The overlap between SNPs with *p* < 10^− 4^ was 3.6% in the original dataset, 16.5% in the 2x dataset and 26.6% in the 4x dataset. In addition, when we randomly allocated cases 10 times in our identification sample, we found the percentage of overlap between the two definitions at *p* < 10^− 4^ varied between 0 to 16%, compared to 4% in the original dataset (see Additional file 1: Table S8).

### Validation of overlapping SNPs

In never-smokers of LifeLines, two SNPs were overlapping between the FEV_1_/FVC < 70% and < LLN definitions at a threshold of p < 10^− 4^ (see Table 3). The first SNP (rs7519348) is located in an intron of the gene nuclear transcription factor Y subunit C (*NFYC*)*,* and the second SNP (rs6913003) is located in an intron of fatty acid binding protein 7 (*FABP7*, see Additional file 1: Figure S4 and S5 for LocusZoom plots). The minor alleles of both SNPs were associated with a higher risk of airflow obstruction and had comparable odds ratios in both analyses. The SNP in *NFYC* (rs7519348) was significantly associated with FEV_1_/FVC < LLN in the SNP validation meta-analysis (*p* = 0.034), but did not pass the multiple testing correction (0.05/2 = 0.025), and was not significantly associated with FEV_1_/FVC < 70% in the SNP validation meta-analysis (*p* = 0.07). The SNP in *FABP7* (rs6913003) was not significantly associated with FEV_1_/FVC < 70% or < LLN in the SNP validation meta-analyses (*p* = 0.08 in both), although the direction of effect was the same in all independent cohorts. Both SNPs did not reach genome-wide significance according to the Bonferroni-corrected threshold (*p* < 2.19 × 10^− 7^) in the discovery analysis (LifeLines) or meta-analysis of both the discovery and SNP validation samples (see Table 3 and Additional file 1: Table S9). Yet, the odds ratios were comparable between all analyses (see Additional file 1: Table S10 and Figure S6). These two overlapping SNPs were not associated with airflow obstruction in ever-smokers and these associations were significantly different between ever- and never-smokers as shown in the interaction analysis (see Additional file 1: Table S11).

**Table 3:** Results of the overlapping SNPs identified in both genome-wide association studies on FEV_1_/FVC < 70% and FEV_1_/FVC < LLN in never- and ever-smokers

						Discovery analysis			SNP validation meta-analysis				Direction of effect in the independent cohorts^a^
SNP	Chr	A1	MAF	Gene	Test	OR	SE	P	OR	SE	P	I^2^	
*Never-smokers*						*(n = 5070)*			*(n = 1966)*				
rs7519348	1	A	33%	*NFYC* (intronic)	<70%	1.36	0.07	4.92 × 10^−6^	1.21	0.11	0.07	0.0	++++ 0
					<LLN	1.37	0.07	2.27 × 10^−5^	1.40	0.16	0.03	0.0	+++++
rs6913003	6	T	4%	*FABP7* (intronic)	< 70%	1.90	0.13	8.99 × 10^−7^	1.48	0.22	0.08	0.0	+++++
					<LLN	1.83	0.14	1.83 × 10^−5^	1.72	0.31	0.08	0.0	+++++
*Ever-smokers*						*(n = 4855)*			*(n = 3134)*				
rs13118083	4	A	45%	*HHIP* (342 kb 5′)	<70%	1.23	0.05	4.36 × 10^−5^	0.97	0.07	0.60	17.0	+ 0 − − +
					<LLN	1.26	0.05	2.27 × 10^−5^	1.05	0.08	0.57	46.7	++ 0 − +
rs7074210	10	G	18%	*ST8SIA6* (62 kb 5′)	<70%	1.35	0.06	3.08 × 10^−6^	1.05	0.08	0.57	64.7	+ − +++
					<LLN	1.33	0.07	3.23 × 10^−5^	0.99	0.10	0.95	64.5	+ − + − +
rs4930390	11	G	24%	*C11orf80* (intronic)	<70%	0.76	0.06	9.40 × 10^−6^	1.02	0.07	0.74	0.0	− + 0 – 0
					<LLN	0.73	0.07	6.68 × 10^−6^	1.01	0.09	0.91	10.1	−++ − −

SNPs were selected based on having a p-value < 10^−4^ in both the discovery analyses on the fixed ratio of 70% and LLN. The logistic regression model of FEV_1_/FVC < 70% was adjusted for sex, age and height, the LLN model was not adjusted. Ever-smoking models were additionally adjusted for pack-years and current-smoking. Discovery sample = LifeLines cohort study, and SNP validation sample = Vlagtwedde-Vlaardingen and RS I to III. A1 = minor allele (effect allele), *MAF* = minor allele frequency, OR = Odds Ratio, SE = standard error and P = *p*-value, I^2^ = heterogeneity measure

^a^ Order: LifeLines, Vlagtwedde-Vlaardingen, and Rotterdam Study I to III. + represents an OR > 1, − represents an OR < 1, and 0 represents is an OR between 0.95 and 1.05 (no effect)

In ever-smokers of LifeLines, three SNPs were overlapping between the two analyses in at *p* < 10^− 4^ (see Table 3). The first SNP (rs13118083) is annotated to hedgehog interacting protein (*HHIP*, 342 kb away), but is located within the long non-coding RNA *LOC105377462* according to the SNP database by NCBI (https://www.ncbi.nlm.nih.gov/SNP/). The second SNP (rs7074210) is located approximately 62 kb from ST8 Alpha-N-Acetyl-Neuraminide Alpha-2,8-Sialyltransferase 6 (*ST8SIA6*), and the last SNP (rs4930390) is annotated to Chromosome 11 Open Reading Frame 80 (*C11orf80*). The minor alleles of the first 2 SNPs were associated with a higher risk of airflow obstruction and the minor allele of rs4930390 with a lower risk. The effect was significantly different between never- and ever-smokers for SNP rs4930390 according to both definitions and for rs7074210 in the FEV_1_/FVC < 70% analyses (see Additional file 1: Table S11). The three SNPs were not replicated in the SNP validation sample (see Table 3 and Additional file 1: Tables S9-S10).

### Gene expression in lung tissue

**Fig. 3:**
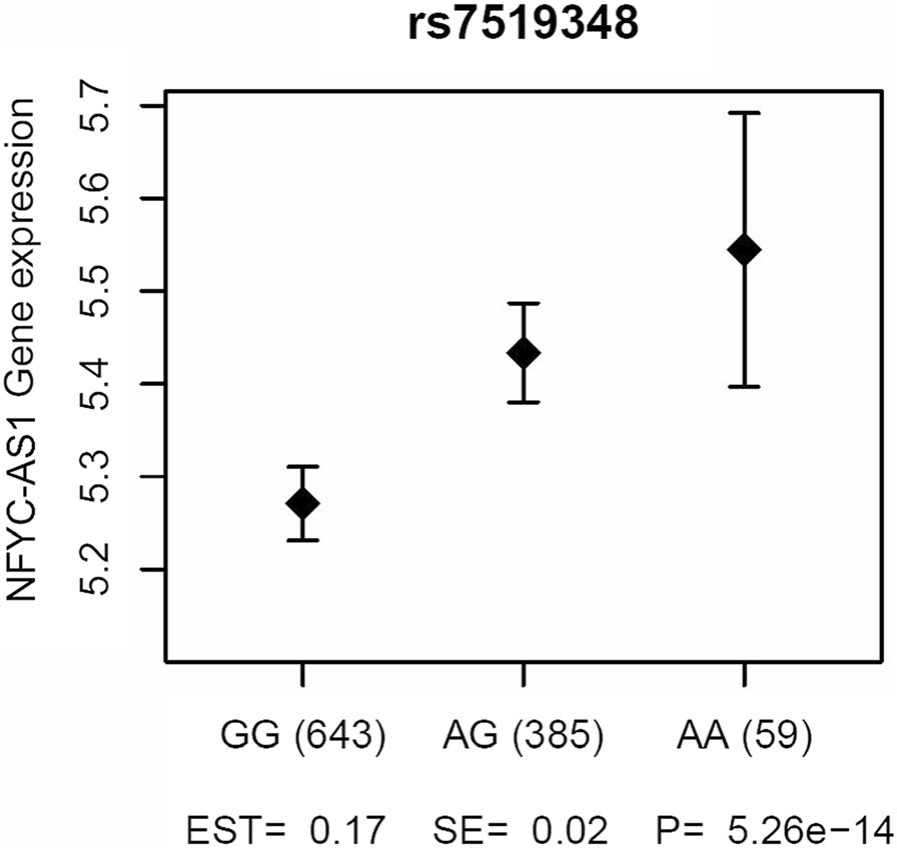
Results of eQTL analysis in lung tissue for rs7519348, an overlapping SNP in never-smokers. The unadjusted mean log2 microarray intensity and 95% CI are plotted, obtained from a meta-analysis of three cohorts included in the lung eQTL dataset

The minor allele (G) of rs7519348 (overlapping SNP in never-smokers) was associated with higher gene expression of *NFYC* Antisense RNA 1 (*NFYC-AS1*) in lung tissue (Fig. 3). Summary statistics of the eQTL analysis for all overlapping SNPs at *p* < 10^− 4^ are provided in Additional file 2.

## Discussion

We investigated the genetic overlap between GWASes using two airflow obstruction definitions in the same population (FEV_1_/FVC < 70 or < LLN). We expected a reasonable overlap in associated SNPs between the two definitions, since 96% of the never-smokers and 93% of the ever-smokers were classified the same way in the discovery sample LifeLines. Surprisingly, only a very small proportion (4% and 6%) of SNPs was overlapping at *p* < 10^− 4^ (see Fig. 1). Even with different significance thresholds the overlap was limited (26% and 29% at *p* < 0.05) (see Table 2). The same observation was made in the replication sample, the Vlagtwedde-Vlaardingen study. In this cohort, 94% and 90% of the never- and ever-smokers, respectively, were classified concordantly, but at p < 0.05 only 24% or 25% of the SNPs were overlapping. In addition, the effect estimates for the two airflow obstruction definitions correlated strongly in both cohorts but the *p*-values showed more variation and correlated only moderately resulting in different top-hits depending on the obstruction definition (see Fig. 2). Thus, the chosen strategy and definition of airflow obstruction had a substantial influence on the GWAS results. This implies that in a discovery-replication design with a predetermined selection p-value, different genetic variants would be followed-up depending on the definition used. In addition, there was no correlation between the p-values nor between the ORs of never- and ever-smokers in both cohorts. None of the selected SNPs overlapped between never- and ever-smokers at p < 10^− 4^, and at *p* < 0.05 the overlap was only 3% in LifeLines (discovery sample) and 2% in Vlagtwedde-Vlaardingen (replication sample, see Table 2). The current study therefore also highlights the importance of stratifying the analysis according to smoking status.

The difference between results from the two definitions might be explained by the fact that obstructive airway diseases are heterogeneous diseases with multiple phenotypes, symptoms and comorbidities. It might thus be beneficial for future GWA studies to focus more on specific COPD subtypes rather than on a broad definition of airflow obstruction or COPD that can be caused by multiple underlying physiologic and genetic mechanisms. In previous GWA studies, in mainly smokers, on classical COPD phenotypes like emphysema and chronic bronchitis, the well-known general COPD genes (*HHIP*, *CHRNA* and *FAM13A*) were consistently identified [27–32]. Perhaps, to identify specific genetic pathways underlying specific COPD phenotypes we should not study the classical COPD phenotypes, but rather clinical COPD subtypes based on symptoms, comorbidities or pathology.

The *CHRNA5/3* and *HHIP* regions were overlapping between six previous GWA studies on airflow obstruction, using different airflow obstruction definitions and populations [11–16]. In the current study, two of the identified SNPs in ever-smokers were located in the *CHRNA5* and *HHIP* regions as well, pointing towards a robust genetic association of these regions with airflow obstruction and COPD (see Additional file 1: Table S6). Likewise, most of previously identified regions associated with airflow obstruction or COPD were nominal significant (*p* < 0.05) in the current study (see Additional file 1: Table S12). Out of the 22 loci identified by the study of Hobbs et al, SNPs in 18 loci were associated with at least one of the airflow definitions at a nominal significance (10 SNPs in never-smokers and 12 SNPs in ever smokers) [14]. In never-smokers, 6 of the 10 SNPs were significantly associated with both definitions and in ever-smokers 7 of the 12 SNPs were significantly associated with both definitions. Some SNPs were significant in both never- and ever-smokers (e.g. *HHIP, PID1* and *THSD4*), while others were either only significant in never-smokers (e.g. *FAM13A, DSP* and *RIN3*) or in ever-smokers (e.g. *CHRNA5, TET2* and *ADGRG6*). In addition, many of the loci previously associated with lung function outcomes (FEV_1_, FVC, and FEV_1_/FVC) were also nominal significant (p < 0.05) in the current study (see Additional file 1: Table S13). Specifically, of the loci reported by Wain et al., 23 out of 28 loci for FEV_1_, 10 out of 17 loci for FVC and 38 out of 51 loci for FEV_1_/FVC were associated with at least one of the airflow definitions at a nominal significance [33]. Lastly, we also checked if the top overlapping SNPs were associated with lung function outcomes in our previous GWA studies on FEV_1_, FEV_1_/FVC and FEF_25–75_ [34, 35]. A SNP annotated to *HHIP* was associated with FEV_1_/FVC and FEF_25–75_ in both never- and ever-smokers (results were replicated) and the *CHRNA5/3* region was only associated with FEV_1_/FVC in ever-smokers. The *NFYC* and *FABP7* regions were associated with FEV_1_/FVC (*p* = 4.40 × 10^− 4^ and *p* = 1.87 × 10^− 4^) in never-smokers, and the *FABP7* SNP was also associated with FEF_25–75_ levels (*p* = 0.026). Interestingly, the *NFYC* region was also overlapping between the current study and the study by Pillai et al. We identified multiple SNPs annotated to *NFYC,* whereas Pillai et al. identified a SNP (rs3767943) in the gene *KCNQ4*, which is located on the right side (3′) of *NFYC* [15]. The *NFYC* region might therefore be an interesting region to further study the underlying mechanisms of its association with airflow obstruction.

A SNP in the intron of *NFYC* and a SNP in *FABP7* were the two overlapping SNPs between the airflow obstruction definitions at *p* < 10^− 4^ in never-smokers and showed the same direction of effect in the five independent cohorts. The minor allele of the SNP in *NFYC* (rs7519348) was associated with a higher risk of airflow obstruction. This gene is a highly conserved transcription factor that is predicted by GeneGlobe to bind promoter regions of 218 genes (see Additional file 1: Table S14) including genes previously associated with lung related outcomes, like *ADORA2B, AKAP9, CD163, ELMOD2, HLA-DPB1, ITPR2, KLF10* and *SERPINA6* [27, 36–42]. In more detail, *HLA-DPB1* is a known COPD gene related to disease severity, *SERPINA6* was associated with emphysema, a deletion in *ADORA2B* was shown to be associated with a decrease in lung fibrosis and pulmonary hypertension, and *ELMOD2* is a candidate gene for familial idiopathic pulmonary fibrosis [27, 36, 39, 40]. The identified SNP was not associated with expression levels of *NFYC* in lung tissue, but was an eQTL for a probeset annotated to *NFYC-AS1.* The function of this specific antisense-RNA, which are generally thought to have a regulatory role, is still unknown.

The minor allele of the SNP in *FABP7* (rs6913003) was also associated with a higher risk of airflow obstruction in never-smokers. This SNP was not associated with the expression of *FABP7* or other genes in lung tissue. *FABP7* is an intracellular lipid-binding protein, involved in long-chain fatty acids transport and cell proliferation [43]. It may be involved in abnormal pulmonary development, since lower expression of *FABP7* was found in patients with congenital cystic adenomatoid malformation [44]. In addition, higher expression of *FABP7* was seen in clear cell renal cell carcinoma and the authors suggested that the gene activates the *ERK* and *STAT3* signalling pathways [45]. *STAT3* was implicated to play a role in pulmonary inflammation and thus *FABP7* might indirectly be involved in airflow obstruction [46].

We were aware of the risk for spurious findings due to the low power of our study and thus we validated our top overlapping SNPs in 4 independent validation cohorts. We furthermore investigated the effect of low power on the overlap between the two definitions by increasing our dataset 2 and 4 times. We found that the percentage of overlap increases when the sample size increases, but still the number of SNPs that do not overlap remains high, i.e. 73.4% when the sample size increased 4-fold. So even when the study power is greatly increased, different SNPs will be found depending on the airflow obstruction definition tested. We also performed a simulation study by 10 times randomly allocating airflow obstruction cases and based on this simulation, we have to conclude that the differences and overlap we found could be chance findings, but that is why we validated the overlapping SNPs in 4 independent validation cohorts.

We only assessed a modest number of SNPs (*n* = 227,981 SNPs) compared to previous large GWAS studies (*n* > 1 million SNPs), since we only included genotyped SNPs to prevent any bias by imputation. The disadvantage of this approach is that we may have a lower genomic coverage. Another limitation of the current study is the use of pre-bronchodilator measurements to define airflow obstruction, which preferably should be based on post-bronchodilator measurements. Especially subjects with asthma could be misclassified as having airflow obstruction, but the results of the overlapping SNPs did not change in a sensitivity analysis excluding asthmatics or adjusting for asthma (see Additional file 1: Table S15). Moreover, only a low number of never-smoking subjects had an FEV_1_/FVC < LLN in the three Rotterdam Study cohorts, but nevertheless results were replicated in these never-smokers. Finally, the “FEV_1_/FVC < 70%” model was adjusted for sex, age and height, but the “FEV_1_/FVC < LLN” model was not adjusted for these variables, since they are included in the LLN calculation. If we do however adjust the “FEV_1_/FVC < LLN” model for these variables, the results do not change. The top SNPs are the same and the correlation between *p*-values for the LLN models adjusted and not adjusted is 0.98. In addition, the reported correlation in never-smokers between the two definitions was 0.48 for p-values and 0.78 for OR. If we use the LLN adjusted model the correlation is 0.48 and 0.79, respectively. This confirms that we used appropriate models to assess the genetic overlap between the two airflow definitions.

## Conclusions

The definition of airflow obstruction and the population under study may be important determinants of which SNPs are associated with airflow obstruction, and thus on which variants are selected for replication. It is therefore important to use the same definition of airflow obstruction in future studies, especially in consortia. In addition, future studies should focus more on specific COPD subtypes and subgroups (e.g. based on smoking status), since there was no overlap in results between never- and ever-smokers, pointing towards possible different underlying mechanisms. Finally, our results suggest that the genes *FABP7* and *NFYC*(-*AS1*) could play a role in the pathogenesis of airflow obstruction in never-smokers.

## Additional files

Additional file 1: Supplementary methods, tables and figures. (DOCX 1790 kb)

Additional file 2: GWAS summary statistics. (XLSX 139809 kb)

## Abbreviations

COPD: Chronic Obstructive Pulmonary Disease
eQTL: Expression quantitative trait locus
*FABP7*: Fatty acid binding protein 7
FEV_1_/FVC: Ratio of forced expiratory volume in one second to forced vital capacity
GWAS: Genome-wide association study
MAF: Minor allele frequency
*NFYC*: Nuclear transcription factor Y subunit C
SNP: Single-nucleotide polymorphism
