# Supplementary material for: Limited overlap in significant hits between genome-wide association studies on two airflow obstruction definitions in the same population
Source: BMC Pulm Med. 2019 Mar 7;19:58. doi: 10.1186/s12890-019-0811-0 (PMC6407273; doi:10.1186/s12890-019-0811-0)
Supplement: Supplementary file 1 — Supplementary methods, tables and figures. (DOCX 1790 kb) [file 12890_2019_811_MOESM1_ESM.docx]

**Supporting file 1**

**Limited overlap in significant hits between genome-wide association studies on two airflow obstruction definitions in the same population.**

**Authors:** Diana A van der Plaat^1,2^, Judith M Vonk^1,2^, Lies Lahousse^3,4^, Kim de Jong^1,2^, Alen Faiz^5,2^, Ivana Nedeljkovic^3^, Najaf Amin^3^, Cleo C van Diemen^6^, Guy G Brusselle^3,4,7^, Yohan Bossé^8^, Corry-Anke Brandsma^5,2^, Ke Hao^9^, Peter D. Paré^10^, Cornelia M van Duijn^3^, Dirkje S Postma^11,2^, H Marike Boezen^1,2^

**Author affiliation**: ^1^University of Groningen, University Medical Center Groningen, Department of Epidemiology, Groningen, the Netherlands. ^2^University of Groningen, University Medical Center Groningen, Groningen Research Institute for Asthma and COPD (GRIAC), Groningen, the Netherlands. ^3^Erasmus Medical Center, Department of Epidemiology, Rotterdam, the Netherlands. ^4^Ghent University Hospital, Department of Respiratory Medicine, Ghent, Belgium. ^5^University of Groningen, University Medical Center Groningen, Department of Pathology and Medical Biology, Groningen, the Netherlands.^6^University of Groningen, University Medical Center Groningen, Department of Genetics, Groningen, the Netherlands. ^7^Erasmus Medical Center, Department of Respiratory Medicine, Rotterdam, the Netherlands. ^8^Institut universitaire de cardiologie et de pneumologie de Québec, Department of Molecular Medicine, Laval University, Québec, Canada. ^9^Merck Research Laboratories, Boston, Massachusetts, United States of America. ^10^The University of British Columbia James Hogg Research Laboratory, St Paul's Hospital, Vancouver, Canada. ^11^University of Groningen, University Medical Center Groningen, Department of Pulmonary Diseases, Groningen, the Netherlands.

**Supplementary methods**

These supplementary methods are similar to the supplementary methods of the van der Plaat et al. J. Allergy Clin. Immunol., 2016*.*[1]

*Cohorts and pulmonary measurements*

Discovery sample: The multi-disciplinary prospective population-based LifeLines cohort study has a three generation design and recruited subjects from the Northern provinces of the Netherlands. It aimed to study the role of exposures, genes and their interaction in the onset of common complex diseases. All subjects provided written informed consent and the study was approved by the Medical Ethics Committee of the University Medical Center Groningen, Groningen, the Netherlands. Spirometry was measured using a Welch Allyn Version 1.6.0.489, PC-based SpiroPerfect with Ca Workstation software.[2]

Replication sample and part of the SNP validation sample: The population-based prospective cohort study Vlagtwedde-Vlaardingen aimed to gain insight into chronic airway diseases. Subjects were followed for 25 years and were recruited from Vlagtwedde (a rural area) and Vlaardingen (an urban area). Written informed consent was provided by all participants and the study protocol was approved by the local university medical hospital ethics committee, University of Groningen, University Medical Center Groningen, The Netherlands. For the current study we used data from the last survey in 1989/1990. FEV_1_ as a percentage of inspiratory vital capacity (IVC) was used instead of FEV_1_/FVC and data were collected by performing a slow inspiratory manoeuvre, using a water-sealed spirometer (Lode instruments, Groningen, the Netherlands).[3]

Part of the SNP validation sample: The prospective population-based Rotterdam Study aimed to investigate chronic diseases in the elderly. Subject aged 55 years and older living in the Ommoord district in the city of Rotterdam in the Netherlands were recruited starting in 1990 (RS I). In 2000, more subjects of 55 years and older were recruited for RS II, and in 2006, subjects of 45 years and older were recruited for the third expansion (RS III). All subjects provided written informed consent and the study was approved by the medical ethics committee of Erasmus University. Spirometric data were collected up to 2009 using a SpiroPro pore-Table pirometer (Erich Jaeger, Hoechberg, Germany), and since 2009 using the Jaeger Masterscreen PFT (Care Fusion, the Netherlands).[4]

In all cohorts, pre-bronchodilator spirometric measurements were performed according to the ATS/ERS criteria.[5]

*Gene expression analysis (eQTL analysis)*

A lung tissue dataset established by the lung eQTL consortium was used to assess if the selected SNPs were cis-acting expression quantitative trait loci (eQTL) in a lung tissue. Lung tissue samples from patients were obtained during their lung resection surgery at the three sites, the University of Groningen (GRN), Laval University (Laval) and University of British Columbia (UBC).[6] Written informed consent was provided by all patients. The ethics committees of the Institut universitaire de cardiologie et de pneumologie de Québec (Laval) and the UBC-Providence Health Care Research Institute Ethics Board (UBC) approved the study, and the study protocol was consistent with the Research Code of the University Medical Center Groningen and Dutch national ethical and professional guidelines.

Gene expression profiles were obtained using a custom Affymetrix array (GEO accession number GPL10379 and GSE23546) and genetic profiles were obtained using Illumina Human1M-Duo BeadChip arrays. In total 1,087 subjects were included in the analysis with no missing data, and probesets located within 4 Mb around the replicated SNPs were selected. First, to calculate cohort specific (GRN, Laval and UBC) principal components (PCs) residuals from linear regression models on log2 transformed gene expression levels (of each probeset separately) were used adjusted for disease status (alpha-1 antitrypsin deficiency, idiopathic pulmonary fibrosis, pulmonary hypertension, cystic fibrosis, other disease), age, sex and smoking status (never/ever/unknown). In the main analysis, 14 PCs for GRN and Laval, and 16 for UBC were included, which explained at least one percent of the total variance. Second, associations between the SNPs and log2 transformed gene expression levels (mRNA level) were assessed separately in each cohort using linear regression adjusted for disease status, age, sex, smoking status and a cohort specific number of PCs. Third, a fixed effects meta-analysis was performed using the effect estimates for the three cohorts weighted by the reciprocal of the estimated variance. SNPs with a p-value below the Bonferroni corrected threshold (p=0.05/number of probesets in 4Mb window) in the meta-analysis were considered significantly associated with gene expression level.

**Supplementary references**

1. van der Plaat DA, de Jong K, Lahousse L, Faiz A, Vonk JM, van Diemen CC, et al. Genome-wide association study on the FEV1/FVC ratio in never-smokers identifies HHIP and FAM13A. J Allergy Clin Immunol. 2017;139:533–40.

2. Stolk RP, Rosmalen JG, Postma DS, de Boer RA, Navis G, Slaets JP, et al. Universal risk factors for multifactorial diseases: LifeLines: a three-generation population-based study. Eur J Epidemiol. 2008;23:67–74.

3. van der Lende R Gezondheidsorganisatie T.N.O. R te G. Epidemiology of chronic non-specific lung disease (chronic Bronchitis). A critical analysis of three field surveys of CNSLD carried out in the Netherlands. Van Gorcum; 1969.

4. Hofman A, Brusselle GG, Darwish Murad S, van Duijn CM, Franco OH, Goedegebure A, et al. The Rotterdam Study: 2016 objectives and design update. Eur J Epidemiol. 2015;30:661–708.

5. Miller MR, Hankinson J, Brusasco V, Burgos F, Casaburi R, Coates A, et al. Standardisation of spirometry. Eur Respir J. 2005;26:319–38.

6. Hao K, Bosse Y, Nickle DC, Pare PD, Postma DS, Laviolette M, et al. Lung eQTLs to help reveal the molecular underpinnings of asthma. PLoS Genet. 2012;8:e1003029.

7. Pruim RJ, Welch RP, Sanna S, Teslovich TM, Chines PS, Gliedt TP, et al. LocusZoom: regional visualization of genome-wide association scan results. Bioinformatics. 2010;26:2336–7.

8. Hobbs BD, de Jong K, Lamontagne M, Bosse Y, Shrine N, Artigas MS, et al. Genetic loci associated with chronic obstructive pulmonary disease overlap with loci for lung function and pulmonary fibrosis. Nat Genet. 2017;49:426–32.

9. Wain L V, Shrine N, Artigas MS, Erzurumluoglu AM, Noyvert B, Bossini-Castillo L, et al. Genome-wide association analyses for lung function and chronic obstructive pulmonary disease identify new loci and potential druggable targets. Nat Genet. 2017;49:416–25. doi:10.1038/ng.3787 [doi].

**Supplementary Tables**

Table S1. FEV_1_/FVC<70% versus FEV_1_/FVC<LLN for never- (A/C) and ever-smokers (B/D) in the discovery sample (LifeLines (A/B)) and replication sample (Vlagtwedde-Vlaardingen (C/D)).

| **A) Never-smokers** | | *FEV_1_/FVC <LLN* | |  |
| --- | --- | --- | --- | --- |
| *LifeLines* |  | **No** | **Yes** | **Total** |
| *FEV_1_/FVC <70%* | No | 4492 (88.6%) | 30 (0.6%) | 4522 (89.2%) |
|  | Yes | 177 (3.5%) | 371 (7.3%) | 548 (10.8%) |
|  | Total | 4669 (92.1%) | 401 (7.9%) | 5070 (100%) |

| **B) Ever-smokers** |  | *FEV_1_/FVC <LLN* | |  |
| --- | --- | --- | --- | --- |
| *LifeLines* |  | **No** | **Yes** | **Total** |
| *FEV_1_/FVC <70%* | No | 3717 (76.6%) | 31 (0.6%) | 3748 (77.2%) |
|  | Yes | 305 (6.3%) | 802 (16.5%) | 1107 (22.8%) |
|  | Total | 4022 (82.8%) | 833 (17.2%) | 4855 (100%) |

| **C) Never-smokers** | | *FEV_1_/FVC <LLN* | |  |
| --- | --- | --- | --- | --- |
| *Vlagtwedde-Vlaardingen* | | **No** | **Yes** | **Total** |
| *FEV_1_/FVC <70%* | No | 355 (82.2%) | 0 (0%) | 355 (82.2%) |
|  | Yes | 28 (6.5%) | 49 (11.3%) | 77 (17.8%) |
|  | Total | 383 (88.7%) | 49 (11.3%) | 432 (100%) |

| **D) Ever-smokers** |  | *FEV_1_/FVC <LLN* | |  |
| --- | --- | --- | --- | --- |
| *Vlagtwedde-Vlaardingen* | | **No** | **Yes** | **Total** |
| *FEV_1_/FVC <70%* | No | 531 (64.5%) | 5 (0.6%) | 536 (65.1%) |
|  | Yes | 80 (9.7%) | 207 (25.2%) | 287 (34.9%) |
|  | Total | 611 (74.2%) | 212 (25.8%) | 823 (100%) |

Table S2. Characteristics of never- and ever-smokers included in the discovery sample (LifeLines) stratified according to airflow obstruction status.

| ***Never-smokers*** | **No airway**  **obstruction** | **<70% &**  **>LLN** | **>70% &**  **<LLN** | **<70% &**  **<LLN** |
| --- | --- | --- | --- | --- |
| N with no missing data | 4,492 | 177 | 30 | 371 |
| Males, N (%) | 1708 (38) | 75 (42) | 6 (20) | 153 (41) |
| Age (years), median (min-max) | 45 (18-89) | 64 (41-85) | 34 (22-41) | 48 (26-85) |
| Height (cm), mean (SD) | 174 (9) | 173 (10) | 175 (9) | 176 (9) |
|  |  |  |  |  |
| ***Ever-smokers*** | **No airway**  **obstruction** | **<70% &**  **>LLN** | **>70% &**  **<LLN** | **<70% &**  **<LLN** |
| N with no missing data | 3,717 | 305 | 31 | 802 |
| Males, N (%) | 1742 (47) | 204 (67) | 5 (16) | 361 (45) |
| Age (years), median (min-max) | 47 (22-83) | 61 (41-85) | 35 (27-43) | 50 (26-83) |
| Height (cm), mean (SD) | 175 (9) | 176 (10) | 172 (8) | 175 (9) |
| Current-smokers, N (%) | 1,590 (43) | 96 (32) | 21 (68) | 464 (58) |
| Pack-years (years), mean (SD) | 16 (10) | 23 (15) | 13 (6) | 22 (13) |

Table S3. All SNPs with a p-value <10^-4^ in the analysis using the definition FEV_1_/FVC<70% in never-smokers of the discovery sample (LifeLines). For comparison, the ORs of these SNPs in the analysis of FEV_1_/FVC < LLN are also presented.

|  | | | **Annotation** |  | *FEV_1_/FVC<70%* | | |  | *FEV_1_/FVC<LLN* | | |
| --- | --- | --- | --- | --- | --- | --- | --- | --- | --- | --- | --- |
| **SNP** | **Chr** | **A1** | **Nearest gene** | **MAF** | **OR** | **SE** | **P** |  | **OR** | **SE** | **P** |
| **rs6913003** | 6 | T | *FABP7* | 4% | 1.90 | 0.13 | 8.99E-07 |  | 1.83 | 0.14 | 1.83E-05 |
| **rs7519348*** | 1 | A | *NFYC* | 33% | 1.36 | 0.07 | 4.92E-06 |  | 1.37 | 0.07 | 2.27E-05 |
| rs11979680*†* | 7 | T | *CLDN3* | 25% | 1.39 | 0.07 | 8.51E-06 |  | 1.27 | 0.08 | 4.26E-03 |
| rs6460055*†* | 7 | C | *CLDN3* | 25% | 1.38 | 0.07 | 9.42E-06 |  | 1.26 | 0.08 | 4.44E-03 |
| rs16861512 | 2 | C | *LINC00276* | 2% | 2.28 | 0.19 | 1.15E-05 |  | 1.95 | 0.21 | 1.25E-03 |
| rs11693055 | 2 | G | *AC104820.2* | 23% | 1.39 | 0.07 | 1.19E-05 |  | 1.35 | 0.08 | 3.04E-04 |
| rs6713632 | 2 | C | *AC104820.2* | 36% | 1.34 | 0.07 | 1.25E-05 |  | 1.27 | 0.07 | 1.36E-03 |
| rs7601 | 15 | C | *PRC1* | 30% | 1.34 | 0.07 | 2.13E-05 |  | 1.29 | 0.08 | 9.19E-04 |
| rs2274956*‡* | 1 | G | *NFYC* | 12% | 1.47 | 0.09 | 2.64E-05 |  | 1.46 | 0.10 | 1.89E-04 |
| rs7302281” | 12 | A | *TMEM132B* | 10% | 0.58 | 0.13 | 2.64E-05 |  | 0.61 | 0.15 | 6.73E-04 |
| rs2744796*‡* | 1 | C | *NFYC* | 12% | 1.46 | 0.09 | 3.04E-05 |  | 1.45 | 0.10 | 2.04E-04 |
| rs12134020*‡* | 1 | G | *NFYC* | 12% | 1.46 | 0.09 | 3.04E-05 |  | 1.45 | 0.10 | 2.04E-04 |
| rs13232536 | 7 | A | *CLDN4* | 25% | 1.36 | 0.07 | 3.06E-05 |  | 1.26 | 0.08 | 4.73E-03 |
| rs6606743 | 12 | G | *LOC105369975* | 49% | 1.32 | 0.07 | 3.16E-05 |  | 1.27 | 0.07 | 1.25E-03 |
| rs965949* | 1 | G | *RIMS3/NFYC-AS1* | 38% | 1.31 | 0.07 | 3.63E-05 |  | 1.31 | 0.07 | 1.91E-04 |
| rs6706397 | 2 | G | *AC104820.2* | 34% | 1.33 | 0.07 | 3.73E-05 |  | 1.31 | 0.08 | 3.85E-04 |
| rs12411778 | 10 | T | *LRRC27* | 5% | 1.69 | 0.13 | 4.01E-05 |  | 1.51 | 0.15 | 4.73E-03 |
| rs335640 | 5 | T | *PDE8B* | 30% | 0.73 | 0.08 | 4.02E-05 |  | 0.77 | 0.08 | 2.15E-03 |
| rs1427767 | 12 | T | *RP11-1028N23.4* | 46% | 1.31 | 0.07 | 4.22E-05 |  | 1.29 | 0.07 | 6.20E-04 |
| rs643394 | 6 | A | *PPP1R14BP5* | 12% | 1.47 | 0.09 | 4.60E-05 |  | 1.37 | 0.10 | 2.17E-03 |
| rs12314806” | 12 | T | *TMEM132B* | 13% | 0.63 | 0.11 | 4.78E-05 |  | 0.64 | 0.13 | 5.22E-04 |
| rs10489167*‡* | 1 | A | *NFYC* | 12% | 1.45 | 0.09 | 5.08E-05 |  | 1.45 | 0.10 | 2.45E-04 |
| rs7960584 | 12 | A | *RP11-1028N23.4* | 46% | 1.31 | 0.07 | 5.44E-05 |  | 1.25 | 0.07 | 2.96E-03 |
| rs2769255* | 1 | T | *KCNQ4* | 35% | 1.31 | 0.07 | 6.22E-05 |  | 1.32 | 0.07 | 1.77E-04 |
| rs2835189 | 21 | G | *RUNX1* | 14% | 0.66 | 0.11 | 6.77E-05 |  | 0.68 | 0.12 | 1.33E-03 |
| rs12425809 | 12 | G | *TMEM132B* | 7% | 0.54 | 0.15 | 7.05E-05 |  | 0.60 | 0.17 | 2.47E-03 |
| rs2493833 | 6 | C | *TAF8* | 38% | 0.75 | 0.07 | 7.67E-05 |  | 0.78 | 0.08 | 2.19E-03 |
| rs6924562 | 6 | T | *RP1-292B18.4* | 48% | 1.30 | 0.07 | 8.05E-05 |  | 1.15 | 0.07 | 5.61E-02 |
| rs471245 | 5 | G | *CDH6* | 41% | 1.30 | 0.07 | 8.85E-05 |  | 1.23 | 0.07 | 5.97E-03 |
| rs5759049 | 22 | G | *PACSIN2* | 9% | 0.58 | 0.14 | 9.01E-05 |  | 0.68 | 0.15 | 9.52E-03 |
| rs4001107 | 7 | C | *HDAC9* | 28% | 1.32 | 0.07 | 9.13E-05 |  | 1.26 | 0.08 | 3.33E-03 |

*The overlapping SNPs are shown in bold.*

**/†/‡ /" LD R^2^ >0.8*

Table S4. All SNPs with a p-value <10^-4^ in the analysis using the definition FEV_1_/FVC<LLN in never-smokers of the discovery sample (LifeLines). For comparison, the ORs of these SNPs in the analysis of FEV_1_/FVC < 70% are also presented.

|  | | | **Annotation** |  | *FEV_1_/FVC<LLN* | | |  | *FEV_1_/FVC<70%* | | |
| --- | --- | --- | --- | --- | --- | --- | --- | --- | --- | --- | --- |
| **SNP** | **Chr** | **A1** | **Nearest gene** | **MAF** | **OR** | **SE** | **P** |  | **OR** | **SE** | **P** |
| rs6942294 | 6 | A | *RP1-20N2.7* | 2% | 2.66 | 0.21 | 3.41E-06 |  | 2.25 | 0.21 | 1.22E-04 |
| rs955785 | 4 | T | *ADGRA3* | 20% | 1.49 | 0.09 | 4.41E-06 |  | 1.18 | 0.08 | 4.30E-02 |
| rs1494507* | 12 | T | *MSRB3/RP11-230G5.2* | 45% | 1.39 | 0.07 | 7.35E-06 |  | 1.25 | 0.07 | 7.17E-04 |
| rs4762099* | 12 | G | *MSRB3/RP11-230G5.2* | 45% | 1.39 | 0.07 | 9.33E-06 |  | 1.25 | 0.07 | 8.88E-04 |
| rs11175785* | 12 | C | *RP11-230G5.2* | 45% | 1.39 | 0.07 | 1.12E-05 |  | 1.26 | 0.07 | 4.35E-04 |
| rs10429063 | 7 | G | *WBSCR17* | 11% | 1.55 | 0.10 | 1.22E-05 |  | 1.32 | 0.10 | 3.54E-03 |
| rs7956325* | 12 | C | *RP11-230G5.2* | 45% | 1.37 | 0.07 | 1.79E-05 |  | 1.24 | 0.07 | 1.42E-03 |
| rs11923454 | 3 | T | *VGLL4* | 20% | 1.45 | 0.09 | 1.79E-05 |  | 1.27 | 0.08 | 2.85E-03 |
| rs17484145 | 7 | C | *FLJ31818* | 10% | 1.57 | 0.11 | 1.81E-05 |  | 1.40 | 0.10 | 6.65E-04 |
| **rs6913003** | 6 | T | *FABP7* | 4% | 1.83 | 0.14 | 1.83E-05 |  | 1.90 | 0.13 | 8.99E-07 |
| **rs7519348** | 1 | A | *NFYC* | 33% | 1.37 | 0.07 | 2.27E-05 |  | 1.36 | 0.07 | 4.92E-06 |
| rs11639310 | 15 | C | *KATNBL1* | 30% | 0.69 | 0.09 | 2.34E-05 |  | 0.82 | 0.07 | 9.07E-03 |
| rs1110240 | 1 | T | *EIF4G3* | 9% | 1.60 | 0.11 | 2.49E-05 |  | 1.43 | 0.10 | 4.64E-04 |
| rs7007301 | 8 | C | *CSMD1* | 8% | 1.62 | 0.11 | 2.49E-05 |  | 1.37 | 0.11 | 4.23E-03 |
| rs10201457*†* | 2 | C | *REEP1* | 43% | 1.35 | 0.07 | 5.55E-05 |  | 1.19 | 0.07 | 9.42E-03 |
| rs3770857 | 2 | G | *CRIM1* | 24% | 1.38 | 0.08 | 6.06E-05 |  | 1.16 | 0.07 | 5.03E-02 |
| rs4869737*‡* | 6 | C | *CCDC170* | 29% | 1.37 | 0.08 | 6.36E-05 |  | 1.30 | 0.07 | 1.96E-04 |
| rs10038211 | 5 | T | *PRR16* | 30% | 1.36 | 0.08 | 7.38E-05 |  | 1.22 | 0.07 | 4.95E-03 |
| rs11128532 | 3 | T | *SLC6A11* | 36% | 1.35 | 0.08 | 7.39E-05 |  | 1.22 | 0.07 | 2.88E-03 |
| rs311579 | 2 | G | *POLR1A* | 33% | 1.35 | 0.08 | 7.96E-05 |  | 1.18 | 0.07 | 1.94E-02 |
| rs6758173*†* | 2 | G | *REEP1* | 40% | 1.34 | 0.07 | 7.98E-05 |  | 1.17 | 0.07 | 2.19E-02 |
| rs1978096 | 7 | T | *AC002463.3.* | 10% | 1.54 | 0.11 | 7.99E-05 |  | 1.38 | 0.10 | 1.60E-03 |
| rs1532401*‡* | 6 | T | *CCDC170* | 29% | 1.36 | 0.08 | 8.09E-05 |  | 1.30 | 0.07 | 1.74E-04 |
| rs17138501 | 6 | C | *LOC105374894* | 8% | 1.58 | 0.12 | 9.27E-05 |  | 1.34 | 0.11 | 7.95E-03 |
| rs10483100*"* | 22 | C | *DGCR2* | 20% | 0.67 | 0.10 | 9.83E-05 |  | 0.75 | 0.09 | 8.82E-04 |
| rs8135308*"* | 22 | G | *DGCR2* | 20% | 0.67 | 0.10 | 9.83E-05 |  | 0.75 | 0.09 | 1.13E-03 |
| rs1399596 | 21 | C | *KCNJ6* | 26% | 1.37 | 0.08 | 9.94E-05 |  | 1.18 | 0.07 | 2.65E-02 |

*The overlapping SNPs are shown in bold.*

**/†/‡ /" LD R^2^ >0.8*

Table S5. All SNPs with p-value <10^-4^ in the analysis using the definition FEV_1_/FVC<70% in ever-smokers of the discovery sample (LifeLines). For comparison, the ORs of these SNPs in the analysis of FEV_1_/FVC < LLN are also presented.

|  | | | **Annotation** |  | *FEV_1_/FVC<70%* | | |  | *FEV_1_/FVC<LLN* | | |
| --- | --- | --- | --- | --- | --- | --- | --- | --- | --- | --- | --- |
| **SNP** | **Chr** | **A1** | **Nearest gene** | **MAF** | **OR** | **SE** | **P** |  | **OR** | **SE** | **P** |
| **rs7074210** | 10 | G | *ST8SIA6* | 18% | 1.35 | 0.06 | 3.08E-06 |  | 1.33 | 0.07 | 3.23E-05 |
| rs11050426 | 12 | C | *TMTC1* | 14% | 0.69 | 0.08 | 3.63E-06 |  | 0.72 | 0.09 | 1.44E-04 |
| **rs4930390** | 11 | G | *C11orf80* | 24% | 0.76 | 0.06 | 9.40E-06 |  | 0.73 | 0.07 | 6.68E-06 |
| rs2032091*†* | 21 | C | *DSCR4* | 41% | 1.25 | 0.05 | 1.37E-05 |  | 1.20 | 0.05 | 1.10E-03 |
| rs1285802 | 14 | A | *CCDC88C* | 19% | 1.31 | 0.06 | 2.10E-05 |  | 1.28 | 0.07 | 2.96E-04 |
| rs941763 | 14 | A | *CCDC88C* | 19% | 1.31 | 0.06 | 2.24E-05 |  | 1.28 | 0.07 | 2.62E-04 |
| rs1328134 | 6 | C | *MRDS1/OFCC1* | 43% | 1.24 | 0.05 | 2.46E-05 |  | 1.24 | 0.06 | 1.19E-04 |
| rs9983399*†* | 21 | T | *DSCR4* | 41% | 1.24 | 0.05 | 2.65E-05 |  | 1.19 | 0.06 | 1.44E-03 |
| rs10494819 | 1 | C | *CAMSAP2* | 48% | 0.81 | 0.05 | 2.89E-05 |  | 0.83 | 0.05 | 1.03E-03 |
| rs163126 | 5 | T | *ARSB* | 18% | 1.31 | 0.06 | 3.17E-05 |  | 1.21 | 0.07 | 6.57E-03 |
| rs12622607 | 2 | T | *NBAS* | 46% | 0.81 | 0.05 | 3.19E-05 |  | 0.83 | 0.06 | 7.25E-04 |
| **rs13118083** | 4 | A | *HHIP** | 45% | 1.23 | 0.05 | 4.36E-05 |  | 1.26 | 0.05 | 2.27E-05 |
| rs4752496 | 10 | G | *LOC105378521* | 33% | 1.24 | 0.05 | 5.16E-05 |  | 1.16 | 0.06 | 9.63E-03 |
| rs2130319 | 3 | A | *C3orf16* | 38% | 0.81 | 0.05 | 5.62E-05 |  | 0.83 | 0.06 | 1.48E-03 |
| rs10504036 | 8 | T | *ZMAT4* | 32% | 0.80 | 0.06 | 5.82E-05 |  | 0.80 | 0.06 | 2.18E-04 |
| rs6531017 | 8 | C | *C8orf79* | 28% | 0.79 | 0.06 | 6.85E-05 |  | 0.80 | 0.06 | 6.15E-04 |
| rs2554399 | 8 | C | *RBM35A* | 13% | 1.33 | 0.07 | 8.34E-05 |  | 1.29 | 0.08 | 9.47E-04 |
| rs17091682 | 2 | G | *TPO* | 3% | 1.70 | 0.14 | 8.44E-05 |  | 1.50 | 0.14 | 4.68E-03 |
| rs9309464 | 2 | G | *EXOC6B* | 19% | 0.77 | 0.07 | 9.25E-05 |  | 0.76 | 0.07 | 1.95E-04 |
| rs1351234 | 3 | C | *STAC* | 13% | 0.72 | 0.08 | 9.42E-05 |  | 0.78 | 0.09 | 3.99E-03 |
| rs4887320 | 15 | A | *NTRK3* | 33% | 0.80 | 0.06 | 9.85E-05 |  | 0.79 | 0.06 | 1.37E-04 |

*The overlapping SNPs are shown in bold.*

** LOC105377462*

*† LD R^2^ >0.8*

Table S6. All SNPs with p-value <10^-4^ in the analysis using the definition FEV_1_/FVC<LLN in ever-smokers of the discovery sample (LifeLines). For comparison, the ORs of these SNPs in the analysis of FEV_1_/FVC < 70% are also presented.

|  | | | **Annotation** |  | *FEV_1_/FVC<LLN* | | |  | *FEV_1_/FVC<70%* | | |
| --- | --- | --- | --- | --- | --- | --- | --- | --- | --- | --- | --- |
| **SNP** | **Chr** | **A1** | **Nearest gene** | **MAF** | **OR** | **SE** | **P** |  | **OR** | **SE** | **P** |
| **rs4930390** | 11 | G | *C11orf80* | 24% | 0.73 | 0.07 | 6.68E-06 |  | 0.76 | 0.06 | 9.40E-06 |
| rs785448 | 15 | A | *TJP1* | 5% | 1.66 | 0.11 | 8.64E-06 |  | 1.48 | 0.11 | 4.03E-04 |
| rs17755080 | 18 | C | *LOC100505817* | 35% | 1.28 | 0.06 | 1.06E-05 |  | 1.21 | 0.05 | 2.66E-04 |
| rs11233182 | 11 | T | *FLJ42102* | 19% | 1.34 | 0.07 | 1.39E-05 |  | 1.20 | 0.06 | 4.91E-03 |
| rs9556352 | 13 | G | *GPC6* | 14% | 1.37 | 0.07 | 2.19E-05 |  | 1.25 | 0.07 | 1.93E-03 |
| **rs13118083** | 4 | A | *HHIP** | 45% | 1.26 | 0.05 | 2.27E-05 |  | 1.23 | 0.05 | 4.36E-05 |
| rs7449021 | 5 | A | *LOC105379168* | 26% | 0.76 | 0.07 | 2.71E-05 |  | 0.80 | 0.06 | 1.95E-04 |
| rs4819314 | 21 | A | *LOC105372825* | 12% | 1.39 | 0.08 | 2.82E-05 |  | 1.27 | 0.08 | 1.82E-03 |
| **rs7074210** | 10 | G | *ST8SIA6* | 18% | 1.33 | 0.07 | 3.23E-05 |  | 1.35 | 0.06 | 3.08E-06 |
| rs11684288 | 2 | T | *CREB1* | 14% | 1.37 | 0.08 | 3.45E-05 |  | 1.21 | 0.07 | 7.46E-03 |
| rs16955758 | 15 | C | *TJP1* | 8% | 1.46 | 0.09 | 4.57E-05 |  | 1.33 | 0.09 | 1.32E-03 |
| rs6493392 | 15 | A | *ATP8B4* | 39% | 0.79 | 0.06 | 4.72E-05 |  | 0.86 | 0.05 | 5.20E-03 |
| rs13235383 | 7 | A | *LOC105375180* | 39% | 1.25 | 0.06 | 4.86E-05 |  | 1.21 | 0.05 | 2.54E-04 |
| rs2134403 | 5 | G | *IRX1* | 44% | 0.79 | 0.06 | 5.38E-05 |  | 0.82 | 0.05 | 2.46E-04 |
| rs11096686 | 2 | C | *FAM49A* | 12% | 1.38 | 0.08 | 5.57E-05 |  | 1.27 | 0.08 | 1.97E-03 |
| rs13398721 | 2 | C | *LOC101928196* | 13% | 1.36 | 0.08 | 5.76E-05 |  | 1.23 | 0.07 | 4.36E-03 |
| rs484262 | 11 | G | *RBM14-RBM4* | 24% | 0.76 | 0.07 | 5.84E-05 |  | 0.84 | 0.06 | 5.62E-03 |
| rs9418809 | 10 | T | *DOCK1* | 22% | 1.30 | 0.06 | 6.00E-05 |  | 1.21 | 0.06 | 1.68E-03 |
| rs6863131 | 5 | G | *CDH9* | 38% | 0.80 | 0.06 | 6.30E-05 |  | 0.83 | 0.05 | 5.69E-04 |
| rs931794 | 15 | G | *HYKK (CHRNA5)* | 32% | 1.26 | 0.06 | 6.35E-05 |  | 1.13 | 0.05 | 2.84E-02 |
| rs4741790 | 9 | G | *CARM1P1* | 40% | 0.80 | 0.06 | 6.43E-05 |  | 0.82 | 0.05 | 2.54E-04 |
| rs1022630 | 6 | T | *LOC100422737* | 41% | 0.80 | 0.06 | 6.50E-05 |  | 0.83 | 0.05 | 3.32E-04 |
| rs1943891 | 18 | T | *LOC100505817* | 19% | 1.31 | 0.07 | 6.95E-05 |  | 1.19 | 0.06 | 5.62E-03 |
| rs288646 | 16 | A | *CDH8* | 14% | 0.71 | 0.09 | 8.81E-05 |  | 0.74 | 0.08 | 1.57E-04 |
| rs1367917 | 8 | C | *LOC101927066* | 25% | 1.28 | 0.06 | 9.07E-05 |  | 1.21 | 0.06 | 1.33E-03 |
| rs1880753 | 17 | G | *ARL17A/CRHR1* | 43% | 1.24 | 0.06 | 9.31E-05 |  | 1.14 | 0.05 | 1.43E-02 |
| rs13041728 | 20 | A | *LOC105372676* | 30% | 1.26 | 0.06 | 9.37E-05 |  | 1.22 | 0.05 | 2.25E-04 |
| rs2006288 | 21 | T | *PDXK* | 16% | 1.33 | 0.07 | 9.43E-05 |  | 1.21 | 0.07 | 6.60E-03 |
| rs1602238 | 4 | C | *LOC105377462* | 25% | 1.28 | 0.06 | 9.53E-05 |  | 1.23 | 0.06 | 7.55E-04 |

** LOC105377462*

*The overlapping SNPs are shown in bold.*

Table S7. Number of SNPs overlapping between the FEV_1_/FVC <70% and <LLN analyses in original, 2x and 4x never-smokers LifeLines datasets.

|  | **Never-smokers**  **original dataset** | | | |  | **Never-smokers**  **2x original dataset** | | |  |  | **Never-smokers**  **4x original dataset** | | | |
| --- | --- | --- | --- | --- | --- | --- | --- | --- | --- | --- | --- | --- | --- | --- |
|  | ***<70%*** | ***<LLN*** | ***Overlap*** | ***%*** |  | ***<70%*** | ***<LLN*** | ***Overlap*** | ***%*** |  | ***<70%*** | ***<LLN*** | ***Overlap*** | ***%*** |
| <0.05 | 11377 | 11475 | 4755 | 26.3 |  | 37991 | 37884 | 19905 | 35.6 |  | 75126 | 74455 | 45146 | 43.2 |
| <0.01 | 2232 | 2197 | 673 | 17.9 |  | 15587 | 15861 | 6963 | 28.4 |  | 45360 | 45062 | 24561 | 37.3 |
| <10^-3^ | 222 | 233 | 54 | 13.5 |  | 4472 | 4485 | 1590 | 21.6 |  | 22907 | 23009 | 10875 | 31.0 |
| <10^-4^ | 31 | 27 | 2 | 3.6 |  | 1323 | 1306 | 373 | 16.5 |  | 11774 | 11901 | 4972 | 26.6 |
| <10^-5^ | 4 | 4 | 0 | 0 |  | 365 | 392 | 91 | 13.7 |  | 6179 | 6145 | 2306 | 23.0 |
| <10^-6^ | 1 | 0 | 0 | 0 |  | 120 | 141 | 25 | 10.6 |  | 3242 | 3210 | 1060 | 19.7 |
| <Bonferroni* | 0 | 0 | 0 | 0 |  | 58 | 80 | 12 | 9.5 |  | 2150 | 2097 | 640 | 17.7 |

*Original dataset: n=5,070; FEV_1_/FVC<70% n=548; <LLN n=401; overlapping cases=371.*

*2x original dataset: n=10,140; FEV_1_/FVC<70% n=1,096; <LLN n=802; overlapping cases=742.*

*4x original dataset: n=20,280; FEV_1_/FVC<70% n=2,192; <LLN n=1,604; overlapping cases=1,484.*

**P<2.19x10^-7^*

Table S8. Range of overlap between the two airflow obstruction definitions based on 10 simulated datasets with randomly assigned cases.

|  | **FEV_1_/FVC <70%** | |  | **FEV_1_/FVC <LLN** | |  | **Overlap** | |  | **% overlap** | |
| --- | --- | --- | --- | --- | --- | --- | --- | --- | --- | --- | --- |
|  | ***min*** | ***max*** |  | ***min*** | ***max*** |  | ***min*** | ***max*** |  | ***min*** | ***max*** |
| <0.05 | 11029 | 11524 |  | 10974 | 11717 |  | 4440 | 4797 |  | 24.89 | 26.01 |
| <0.01 | 2103 | 2303 |  | 2086 | 2377 |  | 634 | 708 |  | 17.05 | 18.95 |
| <10^-3^ | 170 | 253 |  | 166 | 253 |  | 36 | 61 |  | 9.31 | 14.44 |
| <10^-4^ | 8 | 38 |  | 10 | 28 |  | 0 | 7 |  | 0 | 15.91 |
| <10^-5^ | 0 | 8 |  | 0 | 3 |  | 0 | 2 |  | 0 | 50 |
| <10^-6^ | 0 | 1 |  | 0 | 1 |  | 0 | 0 |  | 0 | 0 |
| <Bonferroni* | 0 | 0 |  | 0 | 0 |  | 0 | 0 |  | 0 | 0 |

*We used the LifeLines never-smokers dataset (n=5,070) and randomly assigned 548 FEV_1_/FVC<70% and 401 <LLN cases, with 371 cases overlapping, and repeated this 10 times.*

**P<2.19x10^-7^*

Table S9. Meta-analyses of both the discovery and SNP validation samples of the overlapping SNPs.

| **SNP** | **Chr** | **A1** | **Gene** | **Test** | **OR** | **SE** | **P** | **I^2^** | **Direction of effect**  **in independent**  **cohorts†** |
| --- | --- | --- | --- | --- | --- | --- | --- | --- | --- |
| ***Never-smokers (n=7,036)*** | | |  |  |  | | | |  |
| rs7519348 | 1 | A | *NFYC*  (intronic) | <70% | 1.32 | 0.11 | 1.43x10^-6^ | 0.0 | ++++0 |
|  |  |  |  | <LLN | 1.38 | 0.08 | 2.20x10^-6^ | 0.0 | +++++ |
| rs6913003 | 6 | T | *FABP7*  (intronic) | <70% | 1.78 | 0.06 | 2.58x10^-7^ | 0.0 | +++++ |
|  |  |  |  | <LLN | 1.81 | 0.13 | 3.85x10^-6^ | 0.0 | +++++ |
|  |  |  |  |  |  |  |  |  |  |
| ***Ever-smokers (n=7,989)*** | | |  |  |  | | | |  |
| rs13118083 | 4 | A | *HHIP*  (342kb 5’) | <70% | 1.12 | 0.04 | 3.85x10^-3^ | 67.3 | +0--+ |
|  |  |  |  | <LLN | 1.19 | 0.04 | 1.42x10^-4^ | 57.7 | ++0-+ |
| rs7074210 | 10 | G | *ST8SIA6*  (62kb 5’) | <70% | 1.23 | 0.05 | 5.55x10^-5^ | 72.1 | +-+++ |
|  |  |  |  | <LLN | 1.21 | 0.06 | 6.14x10^-4^ | 71.4 | +-+-+ |
| rs4930390 | 11 | G | *C11orf80*  (intronic) | <70% | 0.86 | 0.05 | 1.77x10^-3^ | 65.5 | -+0-0 |
|  |  |  |  | <LLN | 0.83 | 0.05 | 4.86x10^-4^ | 65.1 | -++-- |

*The logistic regression model of FEV_1_/FVC<70% was adjusted for sex, age and height, the LLN model was not adjusted. Ever-smoking models were additionally adjusted for pack-years and current-smoking. Discovery sample = LifeLines cohort study, and SNP validation sample = Vlagtwedde-Vlaardingen and RS I to III.*

*A1=minor allele (effect allele), MAF=minor allele frequency, OR=Odds Ratio, SE=standard error and P=p-value, I^2^= heterogeneity measure.*

*† Order: LifeLines, Vlagtwedde-Vlaardingen, and Rotterdam Study I to III. + represents an OR >1, – represents an OR<1 , and 0 represents is an OR between 0.95 and 1.05 (no effect).*

Table S10. Associations between the overlapping SNPs and airflow obstruction (FEV_1_/FVC <70% or <LLN) in never- and ever-smokers in the separate SNP validation cohorts.

|  |  |  |  |  | *Vlagtwedde-Vlaardingen* | | | |  | *Rotterdam Study I* | | | |  | *Rotterdam Study II* | | | |  | *Rotterdam Study III* | | | |
| --- | --- | --- | --- | --- | --- | --- | --- | --- | --- | --- | --- | --- | --- | --- | --- | --- | --- | --- | --- | --- | --- | --- | --- |
| **SNP** | **Chr** | **A1** | **Gene** | **Test** | **%** | **OR** | **SE** | **P** |  | **%** | **OR** | **SE** | **P** |  | **%** | **OR** | **SE** | **P** |  | **%** | **OR** | **SE** | **P** |
| ***Never-smokers*** | | |  |  | (n=432) | | | |  | (n=408) | | | |  | (n=379) | | | |  | (n=747) | | | |
| rs7519348 | 1 | A | *NFYC*  (intronic) | <70% | 33% | 1.40 | 0.20 | 0.09 |  | 34% | 1.38 | 0.22 | 0.15 |  | 35% | 1.30 | 0.29 | 0.37 |  | 35% | 0.95 | 0.19 | 0.78 |
|  |  |  |  | <LLN | 33% | 1.29 | 0.22 | 0.25 |  | 34% | 2.36 | 0.48 | 0.07 |  | 35% | 1.51 | 0.48 | 0.39 |  | 35% | 1.28 | 0.30 | 0.41 |
| rs6913003 | 6 | T | *FABP7*  (intronic) | <70% | 4% | 1.71 | 0.45 | 0.23 |  | 5% | 1.97 | 0.46 | 0.14 |  | 5% | 1.22 | 0.62 | 0.75 |  | 6% | 1.22 | 0.35 | 0.58 |
|  |  |  |  | <LLN | 4% | 1.84 | 0.48 | 0.20 |  | 5% | 1.30 | 1.08 | 0.81 |  | 5% | 2.44 | 0.77 | 0.25 |  | 6% | 1.42 | 0.54 | 0.52 |
|  |  |  |  |  |  |  |  |  |  |  |  |  |  |  |  |  |  |  |  |  |  |  |  |
| ***Ever-smokers*** |  |  |  |  | (n=823) | | | |  | (n=640) | | | |  | (n=583) | | | |  | (n=1,088) | | | |
| rs13118083 | 4 | A | *HHIP*  (342kb 5’) | <70% | 46% | 0.96 | 0.12 | 0.74 |  | 46% | 0.90 | 0.14 | 0.47 |  | 46% | 0.80 | 0.16 | 0.14 |  | 47% | 1.13 | 0.12 | 0.30 |
|  |  |  |  | <LLN | 46% | 1.07 | 0.12 | 0.58 |  | 46% | 0.98 | 0.21 | 0.91 |  | 46% | 0.68 | 0.22 | 0.08 |  | 47% | 1.26 | 0.15 | 0.11 |
| rs7074210 | 10 | G | *ST8SIA6*  (62kb 5’) | <70% | 16% | 0.71 | 0.17 | 0.04 |  | 16% | 1.06 | 0.18 | 0.73 |  | 18% | 1.06 | 0.19 | 0.76 |  | 16% | 1.34 | 0.14 | 0.03 |
|  |  |  |  | <LLN | 16% | 0.82 | 0.17 | 0.23 |  | 16% | 1.37 | 0.25 | 0.21 |  | 18% | 0.55 | 0.31 | 0.05 |  | 16% | 1.26 | 0.17 | 0.18 |
| rs4930390 | 11 | G | *C11orf80*  (intronic) | <70% | 24% | 1.15 | 0.14 | 0.33 |  | 26% | 1.05 | 0.15 | 0.75 |  | 25% | 0.87 | 0.17 | 0.41 |  | 26% | 1.01 | 0.13 | 0.97 |
|  |  |  |  | <LLN | 24% | 1.09 | 0.14 | 0.55 |  | 26% | 1.29 | 0.21 | 0.22 |  | 25% | 0.83 | 0.24 | 0.42 |  | 26% | 0.85 | 0.17 | 0.35 |

*%=minor allele frequency (MAF), OR=Odds Ratio, SE=standard error and P=p-value*

Table S11. Analyses stratified according to smoking status in the discovery sample (LifeLines) for the overlapping SNPs.

|  | | |  |  | *Never-smokers* | | |  | *Ever-smokers (>5 py)* | | |  | *SNP*ever-smoking* | | |
| --- | --- | --- | --- | --- | --- | --- | --- | --- | --- | --- | --- | --- | --- | --- | --- |
|  |  |  |  |  | *(n=5,070)* | | |  | *(n=4,855)* | | |  | *interaction (n=9,925)* | | |
| **SNP** | **Chr** | **A1** | **Gene** | **Test** | **OR** | **SE** | **P** |  | **OR** | **SE** | **P** |  | **OR** | **SE** | **P** |
| ***Never-smokers*** | | |  |  |  |  |  |  |  |  |  |  |  |  |  |
| rs7519348 | 1 | A | *NFYC*  (intronic) | <70% | 1.36 | 0.07 | 4.92x10^-6^ |  | 1.01 | 0.06 | 0.92 |  | 0.74 | 0.09 | 4.45x10^-4^ |
|  |  |  |  | <LLN | 1.37 | 0.07 | 2.27x10^-5^ |  | 0.92 | 0.06 | 0.17 |  | 0.67 | 0.10 | 3.68x10^-4^ |
| rs6913003 | 6 | T | *FABP7*  (intronic) | <70% | 1.90 | 0.13 | 8.99x10^-7^ |  | 1.15 | 0.12 | 0.26 |  | 0.59 | 0.18 | 3.12x10^-3^ |
|  |  |  |  | <LLN | 1.83 | 0.14 | 1.83x10^-5^ |  | 1.01 | 0.13 | 0.97 |  | 0.54 | 0.19 | 1.43x10^-3^ |
|  |  |  |  |  |  |  |  |  |  |  |  |  |  |  |  |
| ***Ever-smokers*** | | |  |  |  |  |  |  |  |  |  |  |  |  |  |
| rs13118083 | 4 | A | *HHIP*  (342kb 5’) | <70% | 1.09 | 0.07 | 0.21 |  | 1.23 | 0.05 | 4.36x10^-5^ |  | 1.12 | 0.08 | 1.57x10^-1^ |
|  |  |  |  | <LLN | 1.11 | 0.07 | 0.14 |  | 1.26 | 0.05 | 2.27x10^-5^ |  | 1.11 | 0.09 | 2.39x10^-1^ |
| rs7074210 | 10 | G | *ST8SIA6*  (62kb 5’) | <70% | 1.08 | 0.09 | 0.37 |  | 1.35 | 0.06 | 3.08x10^-6^ |  | 1.25 | 0.11 | 3.87x10^-2^ |
|  |  |  |  | <LLN | 1.11 | 0.10 | 0.27 |  | 1.33 | 0.07 | 3.23x10^-5^ |  | 1.18 | 0.12 | 1.60x10^-1^ |
| rs4930390 | 11 | G | *C11orf80*  (intronic) | <70% | 1.03 | 0.08 | 0.73 |  | 0.76 | 0.06 | 9.40x10^-6^ |  | 0.74 | 0.10 | 2.08x10^-3^ |
|  |  |  |  | <LLN | 0.97 | 0.09 | 0.74 |  | 0.73 | 0.07 | 6.68x10^-6^ |  | 0.75 | 0.11 | 8.61x10^-3^ |

*The logistic regression model of FEV_1_/FVC<70% was adjusted for sex, age and height, the LLN model was not adjusted. The models were additionally adjusted for pack-years (py) and current-smoking in the ever-smokers and the SNP*ever-smoking interaction models.*

Table S12. Associations of SNPs previously identified in the GWAS on COPD by Hobbs et al. [8] in our discovery analyses in LifeLines.

|  |  |  | *FEV_1_/FVC<70% never-smokers* | | |  | *FEV_1_/FVC<LLN never-smokers* | | |  | *FEV_1_/FVC<70% ever-smokers* | | |  | *FEV_1_/FVC<LLN ever-smokers* | | |
| --- | --- | --- | --- | --- | --- | --- | --- | --- | --- | --- | --- | --- | --- | --- | --- | --- | --- |
| **Nearest gene** | **CHR** | **SNP** | **OR** | **SE** | **P** |  | **OR** | **SE** | **P** |  | **OR** | **SE** | **P** |  | **OR** | **SE** | **P** |
| *TGFB2* | 1 | rs12038116 | 1.181 | 0.088 | 0.058 |  | 1.211 | 0.097 | **0.048** |  | 1.022 | 0.070 | 0.758 |  | 0.967 | 0.076 | 0.659 |
| *PID1 (a)* | 2 | rs7578485 | 0.936 | 0.084 | 0.435 |  | 0.915 | 0.095 | 0.350 |  | 0.877 | 0.066 | **0.045** |  | 0.783 | 0.073 | **0.001** |
| *PID1 (b)* | 2 | rs4321351 | 0.838 | 0.074 | **0.018** |  | 0.771 | 0.085 | **0.002** |  | 1.004 | 0.057 | 0.945 |  | 1.007 | 0.061 | 0.911 |
| *RARB* | 3 | rs13087022 | 0.718 | 0.189 | 0.079 |  | 0.729 | 0.210 | 0.131 |  | 1.156 | 0.125 | 0.247 |  | 1.056 | 0.137 | 0.690 |
| *EEFSEC* | 3 | rs2999068 | 0.909 | 0.104 | 0.354 |  | 0.855 | 0.119 | 0.187 |  | 0.886 | 0.083 | 0.144 |  | 0.840 | 0.090 | 0.054 |
| *FAM13A* | 4 | rs6849143 | 1.220 | 0.066 | **0.003** |  | 1.266 | 0.074 | **0.001** |  | 0.984 | 0.052 | 0.761 |  | 1.000 | 0.055 | 0.993 |
| *TET2* | 4 | rs2047409 | 0.966 | 0.069 | 0.615 |  | 0.934 | 0.078 | 0.379 |  | 0.896 | 0.055 | **0.044** |  | 0.881 | 0.059 | **0.032** |
| *GSTCD* | 4 | rs11724839 | 1.056 | 0.147 | 0.713 |  | 0.944 | 0.171 | 0.735 |  | 0.715 | 0.128 | **0.009** |  | 0.782 | 0.136 | 0.070 |
| *HHIP (a)* | 4 | rs1512282 | 0.822 | 0.068 | **0.004** |  | 0.786 | 0.076 | **0.002** |  | 0.868 | 0.052 | **0.007** |  | 0.832 | 0.057 | **0.001** |
| *HHIP (b)* | 4 | rs13118083 | 1.085 | 0.065 | 0.212 |  | 1.114 | 0.073 | 0.137 |  | 1.23 | 0.05 | **4.4E-05** |  | 1.26 | 0.05 | **2.3E-05** |
| *HTR4* | 5 | rs7702840 | 0.891 | 0.169 | 0.497 |  | 0.926 | 0.188 | 0.682 |  | 1.451 | 0.128 | **0.004** |  | 1.167 | 0.140 | 0.271 |
| *ADAM19* | 5 | rs2277027 | 1.169 | 0.069 | **0.025** |  | 1.098 | 0.078 | 0.233 |  | 1.099 | 0.055 | 0.085 |  | 1.096 | 0.059 | 0.120 |
| *DSP* | 6 | rs10484325 | 1.222 | 0.089 | **0.024** |  | 1.214 | 0.099 | **0.049** |  | 1.141 | 0.070 | 0.059 |  | 1.150 | 0.075 | 0.062 |
| *AGER* | 6 | rs2856437 | 0.947 | 0.177 | 0.759 |  | 0.743 | 0.221 | 0.181 |  | 0.958 | 0.134 | 0.747 |  | 0.905 | 0.147 | 0.497 |
| *ARMC2* | 6 | rs1475055 | 1.124 | 0.080 | 0.142 |  | 1.201 | 0.087 | **0.035** |  | 1.161 | 0.063 | **0.018** |  | 1.183 | 0.067 | **0.012** |
| *ADGRG6* | 6 | rs171891 | 0.955 | 0.088 | 0.603 |  | 1.000 | 0.097 | 0.997 |  | 0.807 | 0.070 | **0.002** |  | 0.820 | 0.075 | **0.008** |
| *SFTPD* | 10 | rs1885550 | 1.114 | 0.084 | 0.195 |  | 1.238 | 0.091 | **0.019** |  | 1.042 | 0.067 | 0.537 |  | 1.029 | 0.072 | 0.686 |
| *RIN3* | 14 | rs8008611 | 1.238 | 0.100 | **0.032** |  | 1.301 | 0.109 | **0.015** |  | 1.057 | 0.081 | 0.494 |  | 1.071 | 0.086 | 0.427 |
| *THSD4 (a)* | 15 | rs12899618 | 1.008 | 0.085 | 0.930 |  | 0.968 | 0.095 | 0.731 |  | 1.218 | 0.065 | **0.002** |  | 1.216 | 0.069 | **0.004** |
| *THSD4 (b)* | 15 | rs751998 | 0.813 | 0.076 | **0.006** |  | 0.805 | 0.086 | **0.011** |  | 0.911 | 0.057 | 0.100 |  | 0.937 | 0.060 | 0.283 |
| *CHRNA5* | 15 | rs931794 | 0.959 | 0.071 | 0.554 |  | 1.009 | 0.079 | 0.909 |  | 1.127 | 0.054 | **0.028** |  | 1.260 | 0.058 | **0.0001** |
| *CCDC101* | 16 | rs2008514 | 0.911 | 0.066 | 0.161 |  | 0.934 | 0.074 | 0.356 |  | 1.128 | 0.052 | **0.020** |  | 1.090 | 0.055 | 0.119 |
| *CFDP1* | 16 | rs247447 | 0.961 | 0.093 | 0.668 |  | 0.931 | 0.105 | 0.495 |  | 0.861 | 0.072 | **0.039** |  | 0.869 | 0.078 | 0.070 |
| *MTCL1* | 18 | rs7239976 | 1.074 | 0.259 | 0.784 |  | 1.204 | 0.264 | 0.482 |  | 1.491 | 0.200 | **0.046** |  | 1.325 | 0.212 | 0.185 |
| *CYP2A6* | 19 | rs2644916 | 1.021 | 0.075 | 0.779 |  | 0.992 | 0.083 | 0.918 |  | 1.069 | 0.058 | 0.250 |  | 1.026 | 0.063 | 0.679 |

*The large GWAS by Hobbs et al. [8] used 15,256 COPD cases and 47,936 controls to identify 22 genome-wide significant loci with COPD.[8] We used this publication to assess the effect of these 22 previously identified SNPs in our analyses in the LifeLines Cohort Study. Only SNP rs2047409 (TET2) was available in LifeLines and for the other 21 loci we looked for a SNP in LD with the reported SNP or a SNP in the annotated gene.*

Table S13. Associations of SNPs previously identified in the GWAS on lung function by Wain et al. [9] in our discovery analyses in LifeLines.

|  |  |  | *FEV_1_/FVC<70% never-smokers* | | |  | *FEV_1_/FVC<LLN never-smokers* | | |  | *FEV_1_/FVC<70% ever-smokers* | | |  | *FEV_1_/FVC<LLN ever-smokers* | | |
| --- | --- | --- | --- | --- | --- | --- | --- | --- | --- | --- | --- | --- | --- | --- | --- | --- | --- |
| **Nearest gene** | **CHR** | **SNP** | **OR** | **SE** | **P** |  | **OR** | **SE** | **P** |  | **OR** | **SE** | **P** |  | **OR** | **SE** | **P** |
| ***A) FEV_1_*** |  |  |  |  |  |  |  |  |  |  |  |  |  |  |  |  |  |
| *ENSA* | 1 | rs6587516 | 1.116 | 0.067 | 0.104 |  | 1.077 | 0.076 | 0.328 |  | 1.112 | 0.053 | **0.046** |  | 1.135 | 0.057 | **0.027** |
| *TNS1* | 2 | rs3088214 | 1.186 | 0.068 | **0.012** |  | 1.129 | 0.075 | 0.107 |  | 1.185 | 0.053 | **0.001** |  | 1.190 | 0.057 | **0.002** |
| *MECOM* | 3 | rs2032701 | 1.094 | 0.099 | 0.367 |  | 1.062 | 0.112 | 0.592 |  | 1.225 | 0.076 | **0.008** |  | 1.206 | 0.081 | **0.020** |
| *TET2* | 4 | rs2047409 | 0.966 | 0.069 | 0.615 |  | 0.934 | 0.078 | 0.379 |  | 0.896 | 0.055 | **0.044** |  | 0.881 | 0.059 | **0.032** |
| *GSTCD* | 4 | rs11724839 | 1.056 | 0.147 | 0.713 |  | 0.944 | 0.171 | 0.735 |  | 0.715 | 0.128 | **0.009** |  | 0.782 | 0.136 | 0.070 |
| *HTR4* | 5 | rs7702840 | 0.891 | 0.169 | 0.497 |  | 0.926 | 0.188 | 0.682 |  | 1.451 | 0.128 | **0.004** |  | 1.167 | 0.140 | 0.271 |
| *ABLIM3* | 5 | rs10515625 | 1.322 | 0.110 | **0.011** |  | 1.265 | 0.123 | 0.055 |  | 0.986 | 0.092 | 0.876 |  | 0.932 | 0.100 | 0.479 |
| *ZKSCAN3* | 6 | rs6929812 | 1.139 | 0.066 | **0.046** |  | 1.075 | 0.073 | 0.323 |  | 1.037 | 0.051 | 0.467 |  | 1.068 | 0.054 | 0.225 |
| *HLA-DQB1 (a)* | 6 | rs6928482 | 1.007 | 0.068 | 0.914 |  | 1.001 | 0.076 | 0.994 |  | 0.879 | 0.053 | **0.014** |  | 0.895 | 0.056 | **0.047** |
| *HLA-DQB1 (b)* | 6 | rs2647012 | 0.952 | 0.067 | 0.459 |  | 1.021 | 0.074 | 0.779 |  | 1.109 | 0.052 | **0.045** |  | 1.116 | 0.055 | **0.046** |
| *LOC389602* | 7 | rs6460015 | 0.594 | 0.239 | **0.029** |  | 0.541 | 0.277 | **0.026** |  | 1.065 | 0.147 | 0.667 |  | 0.880 | 0.165 | 0.438 |
| *GLIS3* | 9 | rs2791757 | 1.104 | 0.065 | 0.128 |  | 1.032 | 0.073 | 0.662 |  | 1.125 | 0.051 | **0.022** |  | 1.124 | 0.055 | **0.034** |
| *C10orf11* | 10 | rs2637254 | 1.057 | 0.066 | 0.398 |  | 1.064 | 0.074 | 0.396 |  | 0.866 | 0.052 | **0.006** |  | 0.856 | 0.056 | **0.005** |
| *AHNAK* | 11 | rs11231121 | 1.303 | 0.173 | 0.126 |  | 1.525 | 0.183 | **0.021** |  | 1.101 | 0.144 | 0.503 |  | 1.132 | 0.153 | 0.415 |
| *ME3/PRSS23* | 11 | rs2513008 | 1.069 | 0.073 | 0.354 |  | 1.080 | 0.080 | 0.336 |  | 1.135 | 0.055 | **0.022** |  | 1.149 | 0.059 | **0.019** |
| *CDON/RPUSD4* | 11 | rs561164 | 1.190 | 0.068 | **0.011** |  | 1.212 | 0.075 | **0.011** |  | 0.984 | 0.054 | 0.761 |  | 0.981 | 0.058 | 0.738 |
| *MSRB3* | 12 | rs4762099 | 1.247 | 0.067 | **0.001** |  | 1.389 | 0.074 | **9.3E-06** |  | 0.995 | 0.051 | 0.926 |  | 1.051 | 0.055 | 0.360 |
| *TBX3* | 12 | rs10850382 | 0.890 | 0.073 | 0.110 |  | 0.850 | 0.083 | 0.051 |  | 1.033 | 0.055 | 0.554 |  | 1.076 | 0.059 | 0.212 |
| *RBM19 (a)* | 12 | rs5004600 | 1.115 | 0.114 | 0.339 |  | 1.096 | 0.127 | 0.471 |  | 0.813 | 0.096 | **0.030** |  | 0.801 | 0.104 | **0.033** |
| *RBM19 (b)* | 12 | rs1427767 | 1.311 | 0.066 | **4.2E-05** |  | 1.287 | 0.074 | **0.001** |  | 0.972 | 0.051 | 0.576 |  | 0.938 | 0.055 | 0.248 |
| *TRIP11* | 14 | rs910368 | 1.025 | 0.077 | 0.745 |  | 0.988 | 0.086 | 0.888 |  | 0.990 | 0.060 | 0.872 |  | 0.951 | 0.065 | 0.436 |
| *RIN3* | 14 | rs8008611 | 1.238 | 0.100 | **0.032** |  | 1.301 | 0.109 | **0.015** |  | 1.057 | 0.081 | 0.494 |  | 1.071 | 0.086 | 0.427 |
| *KANSL1* | 17 | rs4383188 | 0.980 | 0.080 | 0.804 |  | 0.920 | 0.090 | 0.355 |  | 1.079 | 0.062 | 0.217 |  | 1.153 | 0.065 | **0.029** |
| *TSEN54* | 17 | rs9899149 | 0.980 | 0.121 | 0.867 |  | 0.988 | 0.134 | 0.928 |  | 1.045 | 0.092 | 0.632 |  | 1.050 | 0.098 | 0.615 |
| *ZGPAT* | 20 | rs1291211 | 1.199 | 0.129 | 0.158 |  | 1.143 | 0.143 | 0.348 |  | 0.992 | 0.105 | 0.937 |  | 0.986 | 0.112 | 0.896 |
| *MICAL3* | 22 | rs424765 | 1.202 | 0.065 | **0.005** |  | 1.190 | 0.073 | **0.017** |  | 1.101 | 0.051 | 0.061 |  | 1.115 | 0.055 | **0.048** |
| *MN1 (a)* | 22 | rs2106973 | 0.915 | 0.067 | 0.184 |  | 0.902 | 0.074 | 0.164 |  | 1.041 | 0.051 | 0.438 |  | 1.031 | 0.055 | 0.582 |
| *MN1 (b)* | 22 | rs926467 | 1.029 | 0.108 | 0.790 |  | 1.140 | 0.114 | 0.253 |  | 1.173 | 0.078 | **0.041** |  | 1.098 | 0.084 | 0.266 |

| ***B) FVC*** |  |  |  |  |  |  |  |  |  |  |  |  |  |  |  |  |  |
| --- | --- | --- | --- | --- | --- | --- | --- | --- | --- | --- | --- | --- | --- | --- | --- | --- | --- |
| *SPAG17/TBX15* | 1 | rs7515420 | 0.994 | 0.066 | 0.933 |  | 0.979 | 0.074 | 0.772 |  | 1.053 | 0.052 | 0.323 |  | 1.028 | 0.056 | 0.623 |
| *EFEMP1* | 2 | rs727878 | 1.009 | 0.068 | 0.891 |  | 0.917 | 0.077 | 0.262 |  | 1.061 | 0.052 | 0.261 |  | 1.020 | 0.056 | 0.723 |
| *SUCLG2* | 3 | rs12489223 | 1.246 | 0.076 | **0.004** |  | 1.229 | 0.085 | **0.015** |  | 0.975 | 0.061 | 0.673 |  | 0.962 | 0.066 | 0.556 |
| *RP11-538P18.2* | 3 | rs10936146 | 1.060 | 0.066 | 0.378 |  | 0.983 | 0.075 | 0.816 |  | 0.999 | 0.051 | 0.989 |  | 0.999 | 0.055 | 0.989 |
| *TARS* | 5 | rs7736988 | 0.968 | 0.082 | 0.697 |  | 0.958 | 0.091 | 0.642 |  | 0.983 | 0.064 | 0.786 |  | 1.028 | 0.068 | 0.683 |
| *ARL15* | 5 | rs172139 | 0.970 | 0.069 | 0.654 |  | 0.931 | 0.077 | 0.351 |  | 1.003 | 0.053 | 0.960 |  | 1.012 | 0.057 | 0.831 |
| *BMP6* | 6 | rs267802 | 1.040 | 0.070 | 0.574 |  | 1.090 | 0.078 | 0.266 |  | 0.915 | 0.057 | 0.118 |  | 0.865 | 0.062 | **0.019** |
| *LHX3* | 9 | rs10858250 | 1.203 | 0.073 | **0.012** |  | 1.229 | 0.081 | **0.011** |  | 0.997 | 0.059 | 0.956 |  | 1.038 | 0.063 | 0.551 |
| *DNLZ* | 9 | rs10870165 | 1.192 | 0.066 | **0.008** |  | 1.277 | 0.074 | **0.001** |  | 0.974 | 0.052 | 0.602 |  | 1.088 | 0.055 | 0.127 |
| *MYPN* | 10 | rs7916821 | 0.942 | 0.066 | 0.366 |  | 0.890 | 0.073 | 0.114 |  | 1.104 | 0.051 | 0.055 |  | 1.125 | 0.055 | **0.033** |
| *HSD17B12* | 11 | rs4755202 | 1.186 | 0.066 | **0.010** |  | 1.156 | 0.074 | **0.048** |  | 0.965 | 0.052 | 0.490 |  | 0.968 | 0.055 | 0.550 |
| *PRDM11* | 11 | rs714215 | 1.117 | 0.073 | 0.131 |  | 1.199 | 0.080 | **0.024** |  | 1.007 | 0.058 | 0.901 |  | 1.056 | 0.062 | 0.377 |
| *CCDC91* | 12 | rs2203088 | 0.900 | 0.067 | 0.114 |  | 0.956 | 0.074 | 0.541 |  | 1.011 | 0.052 | 0.835 |  | 1.008 | 0.055 | 0.884 |
| *TBX3/MED13L* | 12 | rs35458 | 1.140 | 0.137 | 0.340 |  | 1.139 | 0.151 | 0.387 |  | 0.847 | 0.105 | 0.112 |  | 0.722 | 0.120 | **0.006** |
| *WWOX* | 16 | rs7194147 | 1.009 | 0.069 | 0.893 |  | 0.945 | 0.078 | 0.464 |  | 1.028 | 0.053 | 0.603 |  | 1.044 | 0.057 | 0.452 |
| *KCNJ2* | 17 | rs4793323 | 1.116 | 0.065 | 0.093 |  | 1.197 | 0.073 | **0.014** |  | 0.980 | 0.051 | 0.694 |  | 0.967 | 0.055 | 0.541 |
| *CASC20/BMP2* | 20 | rs6054383 | 1.164 | 0.067 | **0.022** |  | 1.096 | 0.074 | 0.219 |  | 1.063 | 0.052 | 0.245 |  | 1.061 | 0.056 | 0.286 |
| ***C) FEV_1_/FVC*** |  |  |  |  |  |  |  |  |  |  |  |  |  |  |  |  |  |
| *MFAP2* | 1 | rs9435732 | 0.870 | 0.083 | 0.093 |  | 0.882 | 0.093 | 0.177 |  | 1.049 | 0.061 | 0.429 |  | 0.995 | 0.065 | 0.935 |
| *LOC101929516* | 1 | rs17513135 | 1.113 | 0.073 | 0.143 |  | 1.104 | 0.082 | 0.225 |  | 1.066 | 0.059 | 0.274 |  | 1.043 | 0.063 | 0.503 |
| *CDC7/TGFBR3* | 1 | rs1192404 | 1.228 | 0.085 | **0.016** |  | 1.307 | 0.094 | **0.004** |  | 1.242 | 0.070 | **0.002** |  | 1.223 | 0.074 | **0.007** |
| *TGFBR3/BRDT2* | 1 | rs10783071 | 1.274 | 0.121 | **0.046** |  | 1.142 | 0.135 | 0.324 |  | 0.864 | 0.105 | 0.164 |  | 0.831 | 0.115 | 0.107 |
| *TGFB2* | 1 | rs993925 | 1.093 | 0.068 | 0.194 |  | 1.129 | 0.076 | 0.112 |  | 0.963 | 0.054 | 0.482 |  | 0.993 | 0.058 | 0.899 |
| *RNU5F-1* | 1 | rs4565719 | 1.079 | 0.068 | 0.265 |  | 1.022 | 0.076 | 0.775 |  | 1.017 | 0.053 | 0.745 |  | 1.012 | 0.056 | 0.837 |
| *CHRM3* | 1 | rs12406493 | 1.057 | 0.066 | 0.402 |  | 1.065 | 0.074 | 0.397 |  | 1.119 | 0.051 | **0.028** |  | 1.081 | 0.055 | 0.154 |
| *KCNS3* | 2 | rs12471785 | 1.079 | 0.074 | 0.302 |  | 1.042 | 0.083 | 0.621 |  | 1.079 | 0.059 | 0.192 |  | 1.080 | 0.063 | 0.216 |
| *PID1* | 2 | rs7578485 | 0.936 | 0.084 | 0.435 |  | 0.915 | 0.095 | 0.350 |  | 0.877 | 0.066 | **0.045** |  | 0.783 | 0.073 | **0.001** |
| *TRAF3IP1/ASB1* | 2 | rs3739070 | 0.764 | 0.134 | **0.045** |  | 0.712 | 0.155 | **0.029** |  | 0.960 | 0.102 | 0.687 |  | 0.949 | 0.109 | 0.634 |
| *HDAC4* | 2 | rs4521068 | 0.915 | 0.083 | 0.286 |  | 0.804 | 0.096 | **0.023** |  | 0.877 | 0.064 | **0.041** |  | 0.942 | 0.068 | 0.376 |
| *RARB* | 3 | rs13087022 | 0.718 | 0.189 | 0.079 |  | 0.729 | 0.210 | 0.131 |  | 1.156 | 0.125 | 0.247 |  | 1.056 | 0.137 | 0.690 |
| *CACNA2D3* | 3 | rs1458978 | 1.305 | 0.070 | **0.000** |  | 1.314 | 0.077 | **0.000** |  | 1.062 | 0.056 | 0.284 |  | 1.110 | 0.059 | 0.079 |
| *EEFSEC* | 3 | rs9849210 | 0.878 | 0.079 | 0.098 |  | 0.888 | 0.088 | 0.177 |  | 0.835 | 0.062 | **0.004** |  | 0.813 | 0.067 | **0.002** |
| *MECOM* | 3 | rs13089423 | 0.836 | 0.084 | **0.034** |  | 0.929 | 0.093 | 0.426 |  | 1.019 | 0.064 | 0.767 |  | 1.027 | 0.069 | 0.702 |
| *FAM13A* | 4 | rs7671167 | 0.835 | 0.067 | **0.007** |  | 0.827 | 0.076 | **0.012** |  | 0.992 | 0.052 | 0.872 |  | 0.968 | 0.056 | 0.566 |
| *FAM13A* | 4 | rs6849143 | 1.220 | 0.066 | **0.003** |  | 1.266 | 0.074 | **0.001** |  | 0.984 | 0.052 | 0.761 |  | 1.000 | 0.055 | 0.993 |
| *NPNT* | 4 | rs4600917 | 0.898 | 0.070 | 0.124 |  | 0.854 | 0.079 | **0.045** |  | 0.866 | 0.055 | **0.008** |  | 0.932 | 0.058 | 0.228 |
| *HHIP* | 4 | rs1512282 | 0.822 | 0.068 | **0.004** |  | 0.786 | 0.076 | **0.002** |  | 0.868 | 0.052 | **0.007** |  | 0.832 | 0.057 | **0.001** |
| *ITGA1* | 5 | rs10038773 | 1.180 | 0.081 | **0.041** |  | 1.197 | 0.090 | **0.045** |  | 1.102 | 0.064 | 0.129 |  | 1.118 | 0.068 | 0.101 |
| *SPATA9* | 5 | rs2548125 | 1.151 | 0.067 | **0.036** |  | 1.194 | 0.074 | **0.017** |  | 1.051 | 0.052 | 0.335 |  | 1.067 | 0.055 | 0.241 |
| *C5orf56* | 5 | rs13165038 | 0.899 | 0.070 | 0.125 |  | 0.834 | 0.079 | **0.022** |  | 1.051 | 0.054 | 0.355 |  | 1.043 | 0.058 | 0.463 |
| *CYFIP2* | 5 | rs10515749 | 1.147 | 0.083 | 0.099 |  | 1.232 | 0.091 | **0.022** |  | 1.070 | 0.067 | 0.319 |  | 1.035 | 0.072 | 0.638 |
| *ADAM19* | 5 | rs2277027 | 1.169 | 0.069 | **0.025** |  | 1.098 | 0.078 | 0.233 |  | 1.099 | 0.055 | 0.085 |  | 1.096 | 0.059 | 0.120 |
| *LST1/NCR3* | 6 | rs2857595 | 1.058 | 0.077 | 0.464 |  | 1.055 | 0.086 | 0.536 |  | 1.021 | 0.061 | 0.738 |  | 1.017 | 0.066 | 0.801 |
| *AGER* | 6 | rs2071279 | 0.943 | 0.075 | 0.436 |  | 0.977 | 0.084 | 0.777 |  | 0.877 | 0.058 | **0.024** |  | 0.893 | 0.063 | 0.069 |
| *KCNQ5* | 6 | rs2840796 | 1.184 | 0.078 | **0.031** |  | 1.149 | 0.088 | 0.115 |  | 1.092 | 0.064 | 0.166 |  | 1.160 | 0.067 | **0.027** |
| *ARMC2* | 6 | rs1475055 | 1.124 | 0.080 | 0.142 |  | 1.201 | 0.087 | **0.035** |  | 1.161 | 0.063 | **0.018** |  | 1.183 | 0.067 | **0.012** |
| *GPR126 (a)* | 6 | rs262121 | 1.010 | 0.074 | 0.891 |  | 0.996 | 0.083 | 0.961 |  | 0.845 | 0.058 | **0.004** |  | 0.850 | 0.063 | **0.009** |
| *GPR126 (b)* | 6 | rs171891 | 0.955 | 0.088 | 0.603 |  | 1.000 | 0.097 | 0.997 |  | 0.807 | 0.070 | **0.002** |  | 0.820 | 0.075 | **0.008** |
| *C1GALT1* | 7 | rs2159191 | 1.095 | 0.069 | 0.187 |  | 1.049 | 0.078 | 0.541 |  | 1.114 | 0.053 | **0.042** |  | 1.133 | 0.057 | **0.028** |
| *ZKSCAN1* | 7 | rs4727443 | 0.832 | 0.071 | **0.009** |  | 0.852 | 0.078 | **0.042** |  | 0.930 | 0.054 | 0.180 |  | 0.863 | 0.058 | **0.012** |
| *PTCH1* | 9 | rs16909859 | 0.793 | 0.124 | 0.062 |  | 0.705 | 0.145 | **0.016** |  | 1.203 | 0.086 | **0.032** |  | 1.170 | 0.093 | 0.092 |
| *ASTN2* | 9 | rs811690 | 1.082 | 0.100 | 0.429 |  | 1.084 | 0.112 | 0.472 |  | 1.176 | 0.076 | **0.032** |  | 1.181 | 0.080 | **0.037** |
| *CDC123* | 10 | rs11257622 | 0.882 | 0.088 | 0.153 |  | 0.754 | 0.103 | **0.006** |  | 0.980 | 0.066 | 0.759 |  | 0.978 | 0.071 | 0.758 |
| *SVIL/KIAA1462* | 10 | rs7917431 | 0.865 | 0.069 | **0.036** |  | 0.867 | 0.077 | 0.063 |  | 1.072 | 0.051 | 0.178 |  | 1.027 | 0.055 | 0.634 |
| *LRP1* | 12 | rs10783815 | 1.117 | 0.068 | 0.104 |  | 1.171 | 0.075 | **0.035** |  | 0.967 | 0.053 | 0.533 |  | 0.958 | 0.057 | 0.451 |
| *FGD6* | 12 | rs7956997 | 1.088 | 0.083 | 0.308 |  | 0.995 | 0.095 | 0.956 |  | 1.088 | 0.066 | 0.204 |  | 1.036 | 0.071 | 0.621 |
| *CCDC38* | 12 | rs11610816 | 1.120 | 0.070 | 0.108 |  | 1.110 | 0.079 | 0.185 |  | 1.008 | 0.056 | 0.881 |  | 1.006 | 0.060 | 0.924 |
| *LINC01467* | 14 | rs6574757 | 1.100 | 0.067 | 0.155 |  | 1.020 | 0.075 | 0.791 |  | 1.052 | 0.051 | 0.327 |  | 1.068 | 0.055 | 0.233 |
| *MGA* | 15 | rs2077361 | 1.120 | 0.088 | 0.198 |  | 1.171 | 0.098 | 0.105 |  | 1.085 | 0.069 | 0.238 |  | 1.070 | 0.074 | 0.363 |
| *THSD4* | 15 | rs12899618 | 1.008 | 0.085 | 0.930 |  | 0.968 | 0.095 | 0.731 |  | 1.218 | 0.065 | **0.002** |  | 1.216 | 0.069 | **0.004** |
| *THSD4* | 15 | rs751998 | 0.813 | 0.076 | **0.006** |  | 0.805 | 0.086 | **0.011** |  | 0.911 | 0.057 | 0.100 |  | 0.937 | 0.060 | 0.283 |
| *SH3GL3* | 15 | rs10520584 | 0.794 | 0.081 | **0.004** |  | 0.881 | 0.088 | 0.152 |  | 0.899 | 0.061 | 0.079 |  | 0.920 | 0.065 | 0.195 |
| *TEKT5* | 16 | rs2355217 | 1.226 | 0.078 | **0.009** |  | 1.356 | 0.084 | **3.1E-04** |  | 1.114 | 0.064 | 0.092 |  | 1.090 | 0.068 | 0.207 |
| *MMP15* | 16 | rs2270919 | 1.247 | 0.156 | 0.157 |  | 1.418 | 0.168 | **0.037** |  | 1.396 | 0.116 | **0.004** |  | 1.214 | 0.125 | 0.119 |
| *CFDP1* | 16 | rs247447 | 0.961 | 0.093 | 0.668 |  | 0.931 | 0.105 | 0.495 |  | 0.861 | 0.072 | **0.039** |  | 0.869 | 0.078 | 0.070 |
| *EFCAB5* | 17 | rs9897794 | 0.916 | 0.065 | 0.180 |  | 0.843 | 0.073 | **0.020** |  | 1.048 | 0.051 | 0.362 |  | 0.989 | 0.055 | 0.835 |
| *CISD3* | 17 | rs12451380 | 0.774 | 0.097 | **0.008** |  | 0.650 | 0.117 | **2.3E-04** |  | 1.141 | 0.071 | 0.064 |  | 1.097 | 0.076 | 0.220 |
| *LTBP4* | 19 | rs7259237 | 1.047 | 0.068 | 0.496 |  | 1.090 | 0.076 | 0.257 |  | 1.033 | 0.054 | 0.539 |  | 1.060 | 0.057 | 0.310 |
| *KCNE2* | 21 | rs2834440 | 0.964 | 0.067 | 0.578 |  | 0.970 | 0.074 | 0.682 |  | 1.039 | 0.053 | 0.470 |  | 1.022 | 0.056 | 0.705 |

*The large GWAS by Wain et al. [9] used 48,943 subjects to identify 97 genome-wide significant loci with lung function outcomes (FEV_1_, FVC, and FEV_1_/FVC).[9] We used this publication to assess the effect of these 97 previously identified SNPs in our analyses in the LifeLines Cohort Study. Data for 7 of these SNPs were available in LifeLines (rs2637254, rs17513135, rs1192404, rs993925, rs2857595, rs16909859, and rs2834440) and for the other loci we looked for a SNP in LD with the reported SNP or a SNP in the annotated gene. No proxy could be identified for SNP rs7050036 (AP1S2) on chromosome X.*

Table S14. 218 predicted target genes for NFYC based on GeneGlobe (Aug-2016)

| Predicted targets *NFYC* | | | | | |
| --- | --- | --- | --- | --- | --- |
| *ABI3* | *FADS1* | *LOC100289350* | *RAB37* | | *TSSC1* |
| *ACSL3* | *FADS2* | *LOC440181* | *RAB40B* | | *TTK* |
| *ADNP2* | *FAM118A* | *LOC650024* | *RAD23B* | | *TTTY14* |
| *ADORA2B* | *FAM151A* | *LOC728470* | *RALGPS1* | | *TXN2* |
| *AGBL5* | *FAM21D* | *LOC729770* | *RBM15B* | | *TXNDC2* |
| *AGPAT5* | *FAM3C* | *LOC729900* | *RBM46* | | *UBAP1* |
| *AKAP9* | *FAR1* | *LRP2BP* | *RCL1* | | *UFD1L* |
| *ALPK2* | *FKBP14* | *LSM14B* | *RFX1* | | *UGT2B15* |
| *ALS2CR12* | *FLG2* | *MAEA* | *RIOK3* | | *USP43* |
| *ANKRD37* | *FLJ37786* | *MAGEA1* | *RNASE9* | | *VPS36* |
| *ANKRD54* | *FMO6P* | *MAPRE1* | *RNF216* | | *WDR19* |
| *ANKRD55* | *FN3KRP* | *MARK3* | *ROBO3* | | *WDR62* |
| *AP2S1* | *G6PC2* | *MBD3* | *RPA1* | | *WDR89* |
| *BCAR3* | *GCSH* | *MEGF9* | *RPS6KA2* | | *ZIK1* |
| *BEST1* | *GLRA3* | *MLEC* | *RPSA* | | *ZNF416* |
| *BFAR* | *GTF2I* | *MORF4L2* | *RWDD3* | | *ZNF513* |
| *BICD2* | *GUCA2B* | *MRPL51* | *SACM1L* | | *ZNF836* |
| *BRD8* | *H2AFV* | *MSL1* | *SCARNA5* | | *ZSCAN16* |
| *BSN* | *HCFC1* | *MTHFD1* | *SEC31A* | |  |
| *BTBD8* | *HIST1H1A* | *MUCL1* | *SEL1L2* | |  |
| *C10orf95* | *HIST1H4A* | *NAP1L4* | *SERPINA6* | |  |
| *C12orf49* | *HIST2H2BF* | *NCAPD2* | *SETD5* | |  |
| *C16orf46* | *HIST3H3* | *NFE2L1* | *SLC45A2* | |  |
| *CAPSL* | *HLA-DPB2* | *NIPAL1* | *SMYD4* | |  |
| *CD163* | *HLA-DRB1* | *NIPAL3* | *SNORA23* | |  |
| *CDCA4* | *HMGB3* | *NLRP4* | *SNORA6* | |  |
| *CDKN3* | *HNRNPAB* | *NMS* | *SNORA62* | |  |
| *CELA1* | *HSPB8* | *NMT1* | *SNRNP27* | |  |
| *CEP152* | *HYLS1* | *NOL12* | *SNRPF* | |  |
| *CEP78* | *IDE* | *NUDCD2* | *SPC25* | |  |
| *CHFR* | *IGDCC4* | *ODAM* | *SPDYC* | |  |
| *CHST7* | *INA* | *OS9* | *STAG3L1* | |  |
| *CKAP2* | *IRGQ* | *OSBP* | *STAG3L2* | |  |
| *CNGA1* | *ITPR2* | *OSBPL2* | *STAG3L3* | |  |
| *COPS3* | *KCTD18* | *PARD6B* | *STRA8* | |  |
| *COPZ2* | *KDM3A* | *PDIA4* | *TAF4* | |  |
| *COQ3* | *KIF11* | *PHF14* | *TBX19* | |  |
| *CXorf67* | *KIF21B* | *PIN1* | *TCOF1* | |  |
| *DCP2* | *KLF10* | *PIWIL1* | *TDP1* | |  |
| *DLAT* | *KNCN* | *PKD1L1* | *TERF1* | |  |
| *DNAH6* | *KPNA4* | *PLAC8* | *THAP9* | |  |
| *DNAJB11* | *KPNB1* | *PLBD2* | *TLE3* | |  |
| *DNAJC8* | *KRTAP20-1* | *PLD3* | *TMEM107* | |  |
| *DOK6* | *KRTAP20-4* | *PNRC1* | *TMEM167A* | |  |
| *DSCR3* | *KRTAP6-1* | *POLA1* | *TMEM201* | |  |
| *DYNC1H1* | *KRTAP6-2* | *POLQ* | *TMEM45A* | |  |
| *EIF3L* | *KRTAP9-4* | *POU3F4* | *TMEM50B* |  | |
| *ELMOD2* | *KRTAP9-8* | *PPIC* | *TOB2* | |  |
| *ERCC8* | *LANCL1* | *PPP2R1A* | *TP53TG5* | |  |
| *ERLIN1* | *LARGE* | *PRRT3* | *TPCN1* | |  |

Table S15. Associations of the overlapping SNPs with airflow obstruction (FEV_1_/FVC <70% or <LLN) in all never-/ ever-smokers or never-/ ever-smokers without asthma in the discovery sample Lifelines, in the basic model and models additionally adjusted for asthma defined as doctor diagnosed or having both two or more symptoms and using asthma medication.

| ***Never-smokers*** | | *All never-smokers (n=5,070)*  *Basic model* | | |  | *Never-smokers without*  *asthma (n=4,550)*  *Basic model* | | |  | *All never-smokers (n=5,070)*  *Basic model + asthma adjustment* | | |
| --- | --- | --- | --- | --- | --- | --- | --- | --- | --- | --- | --- | --- |
| **SNP** | **Test** | **OR** | **SE** | **P** |  | **OR** | **SE** | **P** |  | **OR** | **SE** | **P** |
| rs7519348 | <70% | 1.36 | 0.14 | 4.92x10^-6^ |  | 1.35 | 0.08 | 9.52x10^-5^ |  | 1.38 | 0.07 | 4.43x10^-6^ |
|  | <LLN | 1.37 | 0.07 | 2.27x10^-5^ |  | 1.36 | 0.09 | 5.52x10^-4^ |  | 1.37 | 0.08 | 4.68x10^-5^ |
| rs6913003 | <70% | 1.90 | 0.13 | 8.99x10^-7^ |  | 1.93 | 0.15 | 9.78x10^-6^ |  | 1.85 | 0.14 | 6.51x10^-6^ |
|  | <LLN | 1.83 | 0.14 | 1.83x10^-5^ |  | 1.86 | 0.16 | 1.46x10^-4^ |  | 1.79 | 0.15 | 6.79x10^-5^ |

| ***Ever-smokers*** | | *All ever-smokers (n=4,855)*  *Basic model* | | |  | *Ever-smokers without*  *asthma (n=4,448) Basic model* | | |  | *All ever-smokers (n=4,855)*  *Basic model + asthma adjustment* | | |
| --- | --- | --- | --- | --- | --- | --- | --- | --- | --- | --- | --- | --- |
| **SNP** | **Test** | **OR** | **SE** | **P** |  | **OR** | **SE** | **P** |  | **OR** | **SE** | **P** |
| rs13118083 | <70% | 1.23 | 0.05 | 4.36x10^-5^ |  | 1.23 | 0.06 | 2.10x10^-4^ |  | 1.23 | 0.05 | 9.06x10^-5^ |
|  | <LLN | 1.26 | 0.05 | 2.27x10^-5^ |  | 1.25 | 0.06 | 1.44x10^-4^ |  | 1.25 | 0.06 | 6.12x10^-5^ |
| rs7074210 | <70% | 1.35 | 0.06 | 3.08x10^-6^ |  | 1.39 | 0.07 | 1.32x10^-6^ |  | 1.35 | 0.07 | 3.40x10^-6^ |
|  | <LLN | 1.33 | 0.07 | 3.23x10^-5^ |  | 1.36 | 0.07 | 2.35x10^-5^ |  | 1.33 | 0.07 | 3.96x10^-5^ |
| rs4930390 | <70% | 0.76 | 0.06 | 9.40x10^-6^ |  | 0.77 | 0.07 | 8.57x10^-5^ |  | 0.77 | 0.06 | 3.86x10^-5^ |
|  | <LLN | 0.73 | 0.07 | 6.68x10^-6^ |  | 0.73 | 0.08 | 2.38x10^-5^ |  | 0.75 | 0.07 | 2.42x10^-5^ |

**basic models: FEV_1_/FVC <70% ~ SNP + age + sex + height FEV_1_/FVC <LLN ~ SNP (Ever-smoking models were additionally adjusted for pack-years and current-smoking)*

*Asthma was defined as doctor diagnosed, or the use of asthma medication and the presence of >2 asthma symptoms, i.e. wheeze without having a cold, attacks of dyspnea at rest and woken by an attack of dyspnea.*

**Supplementary Figures**


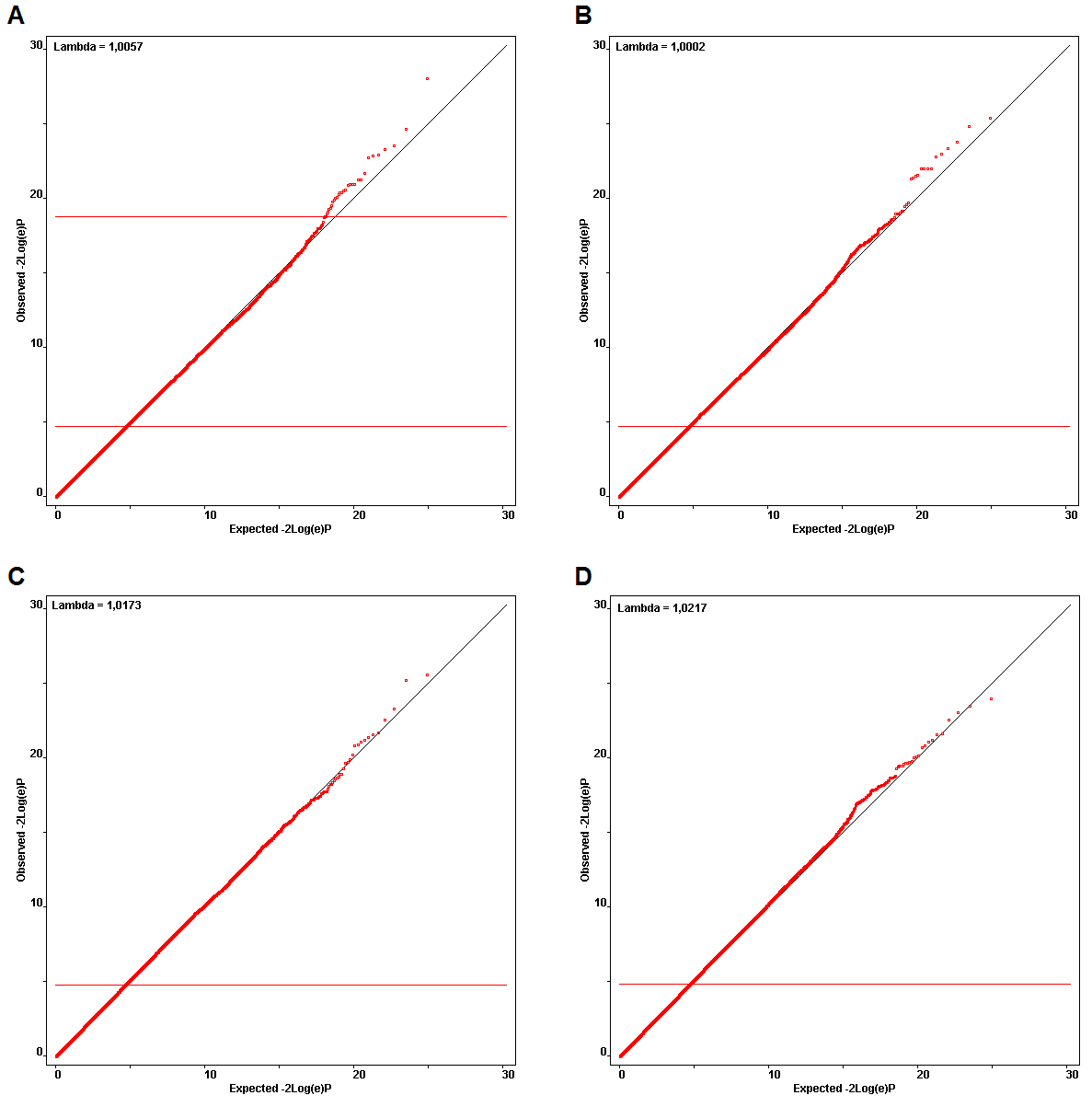


Figure S1. Q-Q plots showing the distribution of observed p-values relative to the expected p-values in the discovery analysis in LifeLines. A) analysis on FEV_1_/FVC <70% in never-smokers, B) analysis on FEV_1_/FVC <LLN in never-smokers, C) analysis on FEV_1_/FVC <70% in ever-smokers, and D) analysis on FEV_1_/FVC <LLN in ever-smokers. All plots show an estimated inflation factor (λ) of 1.0, indicating no significant overall effect of population stratification.


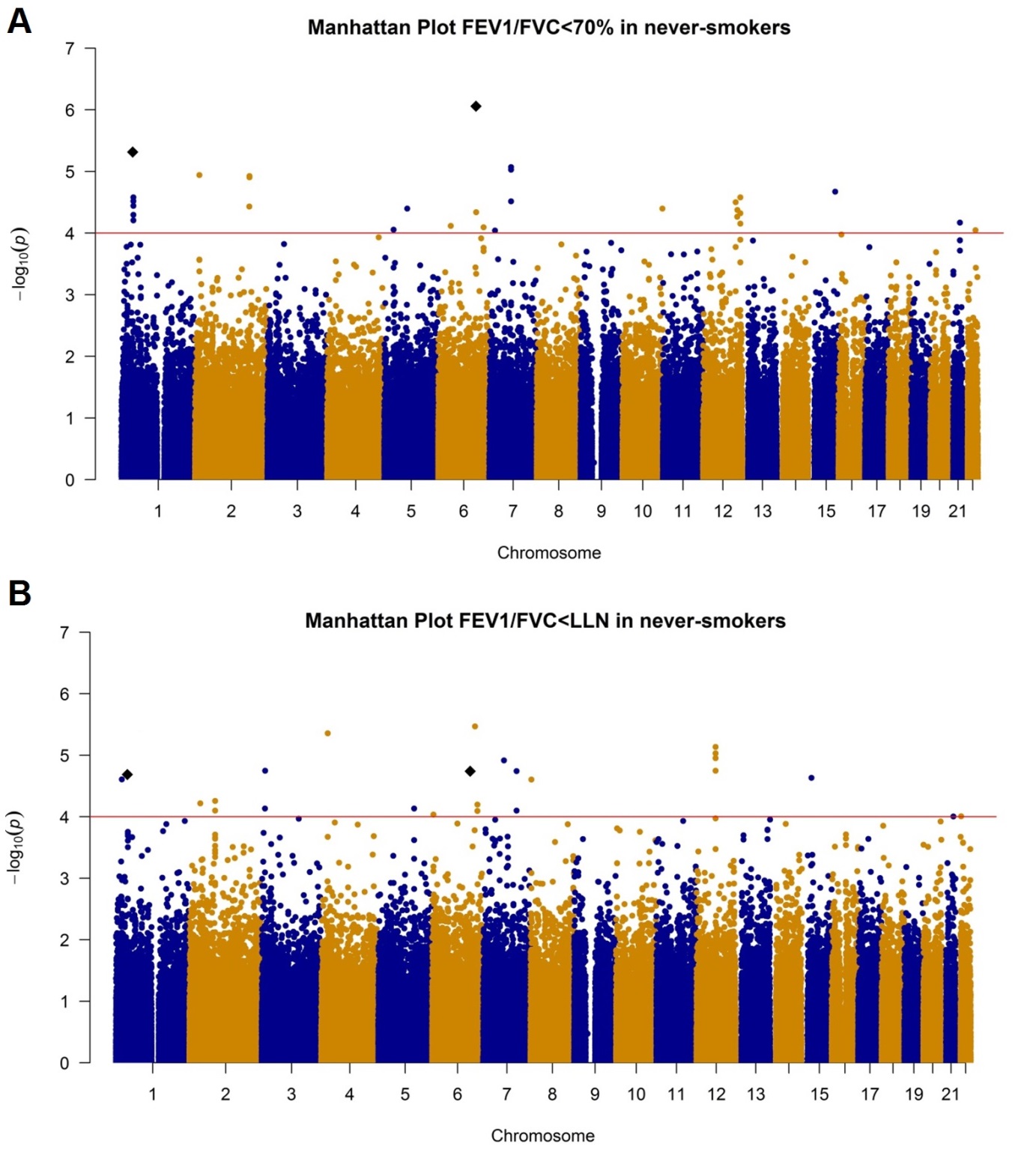


Figure S2. Manhattan plot showing associations between SNPs and airflow obstruction defined as either FEV_1_/FVC<70% (A) or FEV_1_/FVC<LLN (B) in never-smokers of the discovery sample LifeLines. On the x-axis the chromosomal position of the SNPs are shown and on the y-axis the corresponding (negative log10) p-values. The line represents the selection p-value of 10^-4^ and the black diamonds the overlapping SNPs.


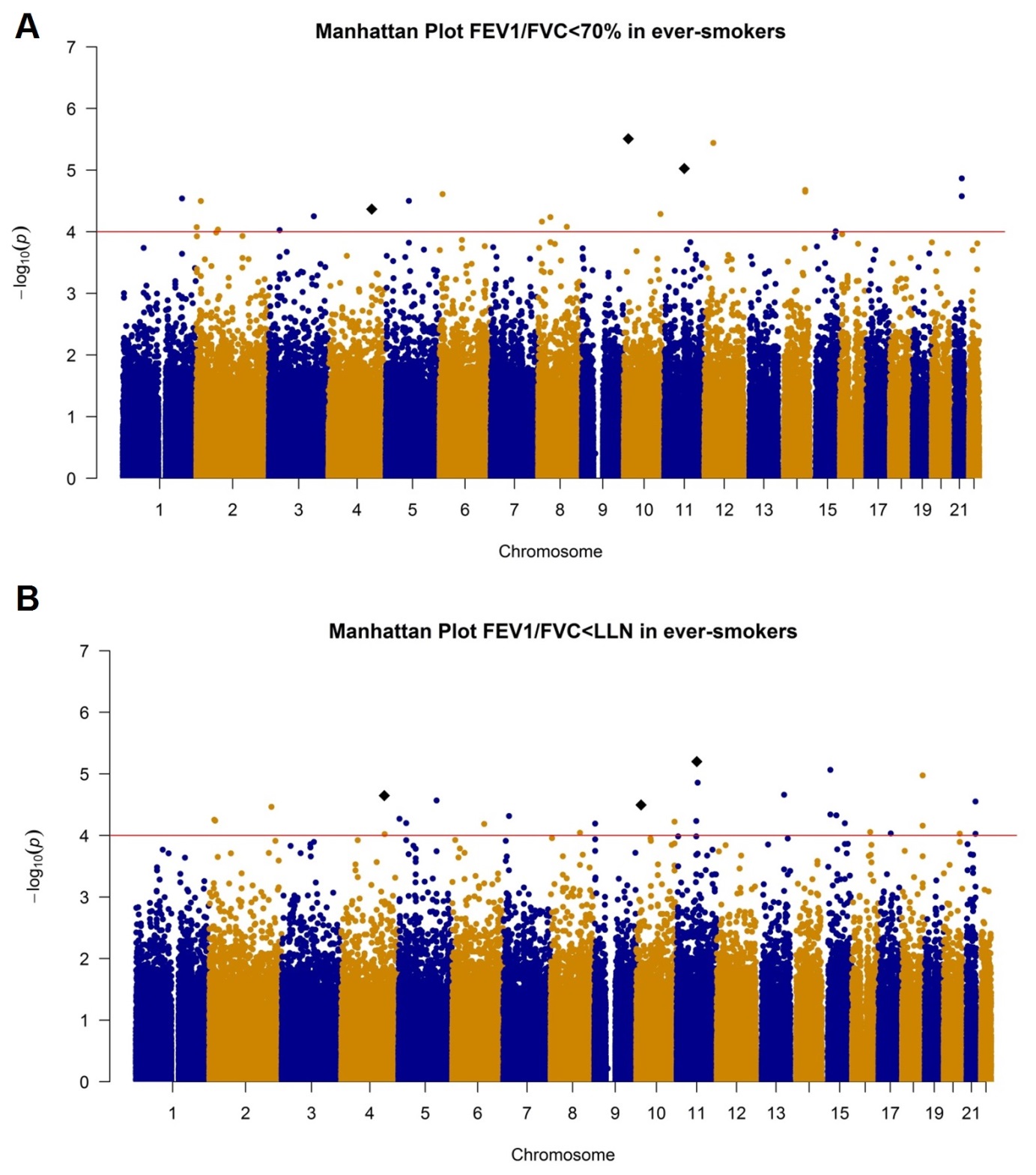


Figure S3. Manhattan plot showing associations between SNPs and airflow obstruction defined as either FEV_1_/FVC<70% (A) or FEV_1_/FVC<LLN (B) in ever-smokers of the discovery sample LifeLines. On the x-axis the chromosomal position of the SNPs are shown and on the y-axis the corresponding (negative log10) p-values. The line represents the selection p-value of 10^-4^ and the black diamonds the overlapping SNPs.


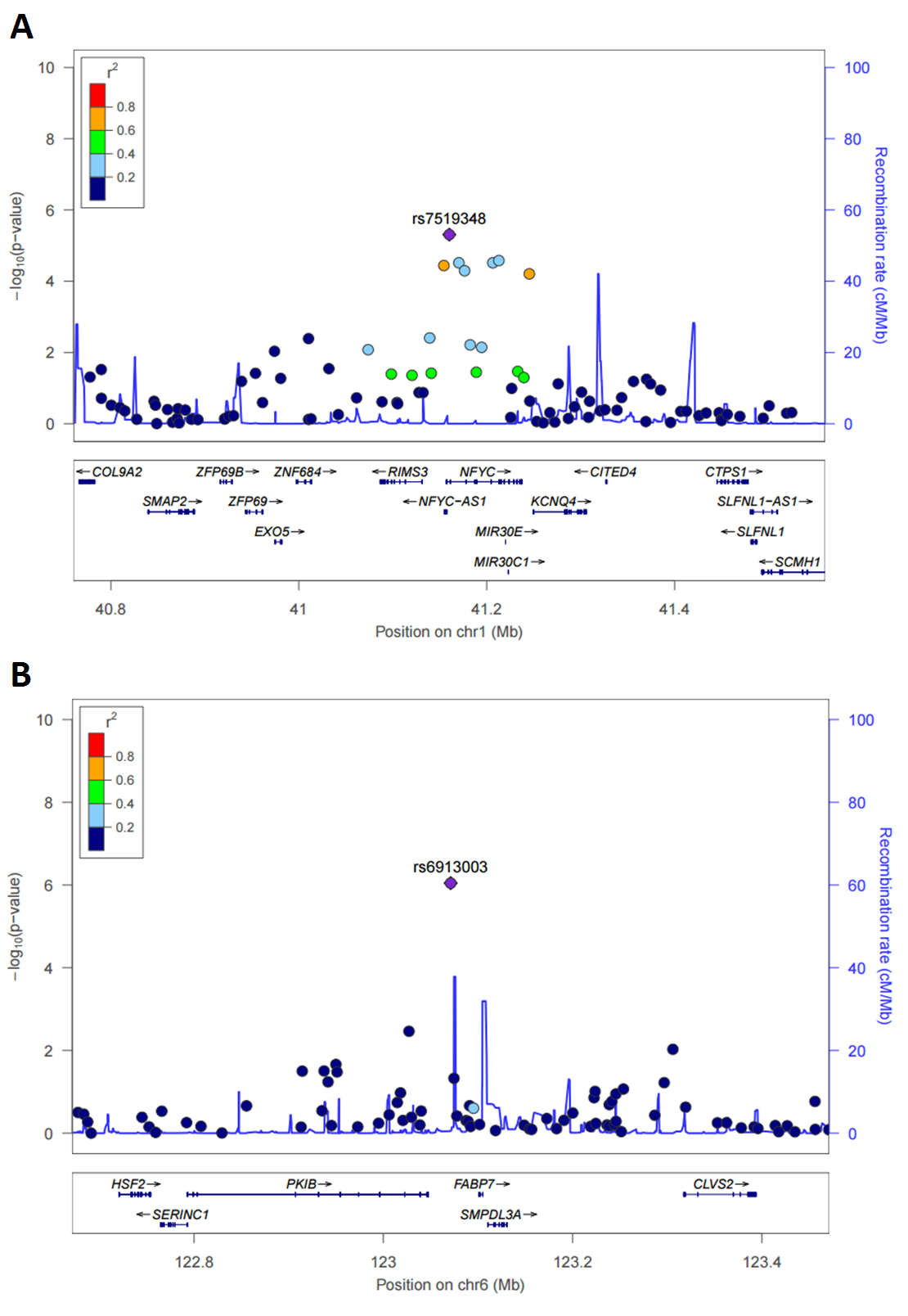


Figure S4. LocusZoom plots for A) *NFYC* (rs7519348) and B) *FABP7* (rs6913003) in the FEV_1_/FVC<70% GWAS in never-smokers of the discovery sample LifeLines.[7] x-axis = megabase (Mb) position on the chromosome, y-axis = negative log10 of the p-values, purple diamond = selected SNP, and see inset legend for the linkage disequilibrium (r^2^) explanation.


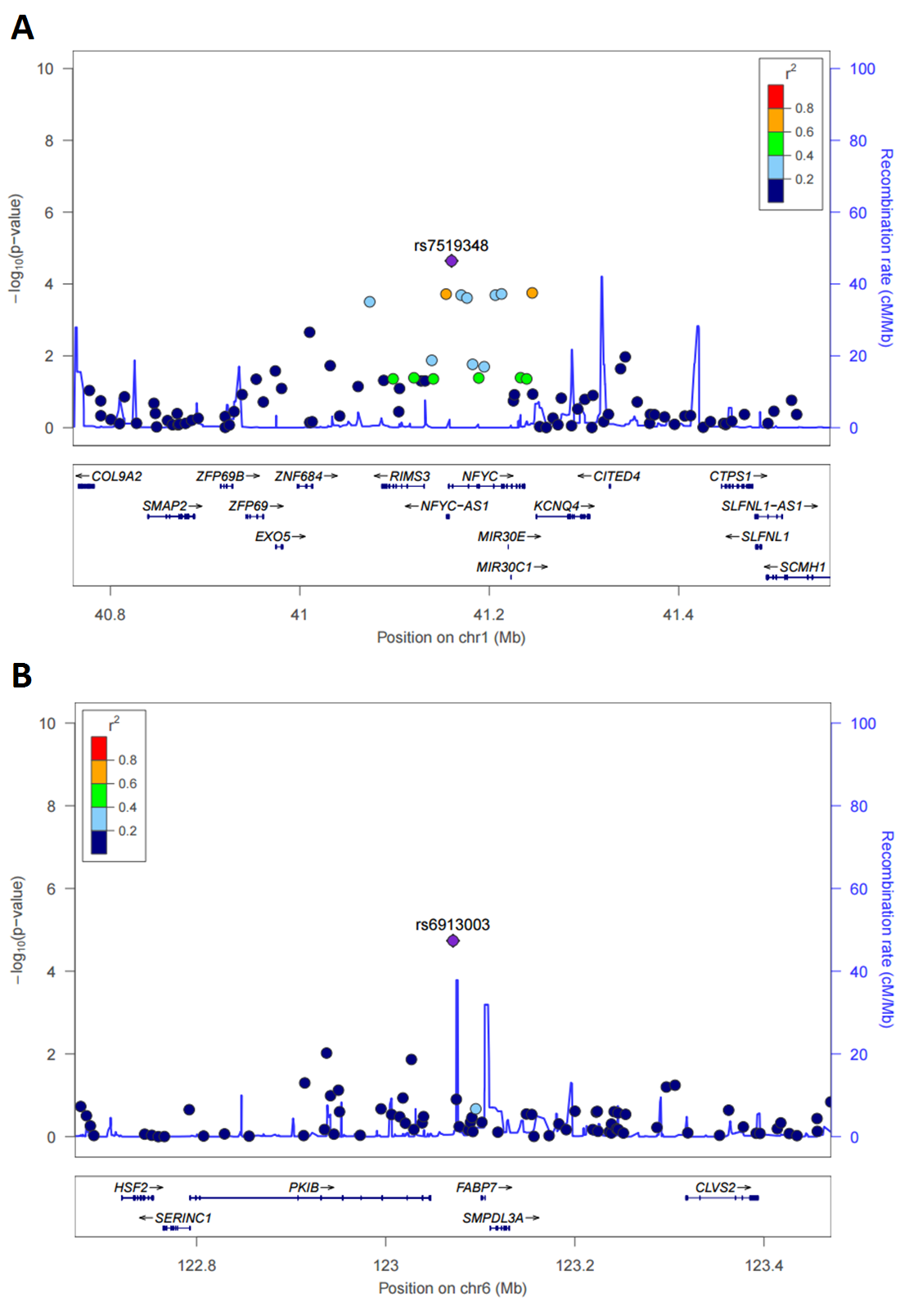


Figure S5. LocusZoom plots for A) *NFYC* (rs7519348) and B) *FABP7* (rs6913003) in the FEV_1_/FVC<LLN GWAS in never-smokers of the discovery sample LifeLines.[7] x-axis = megabase (Mb) position on the chromosome, y-axis = negative log10 of the p-values, purple diamond = selected SNP, and see inset legend for the linkage disequilibrium (r^2^) explanation.


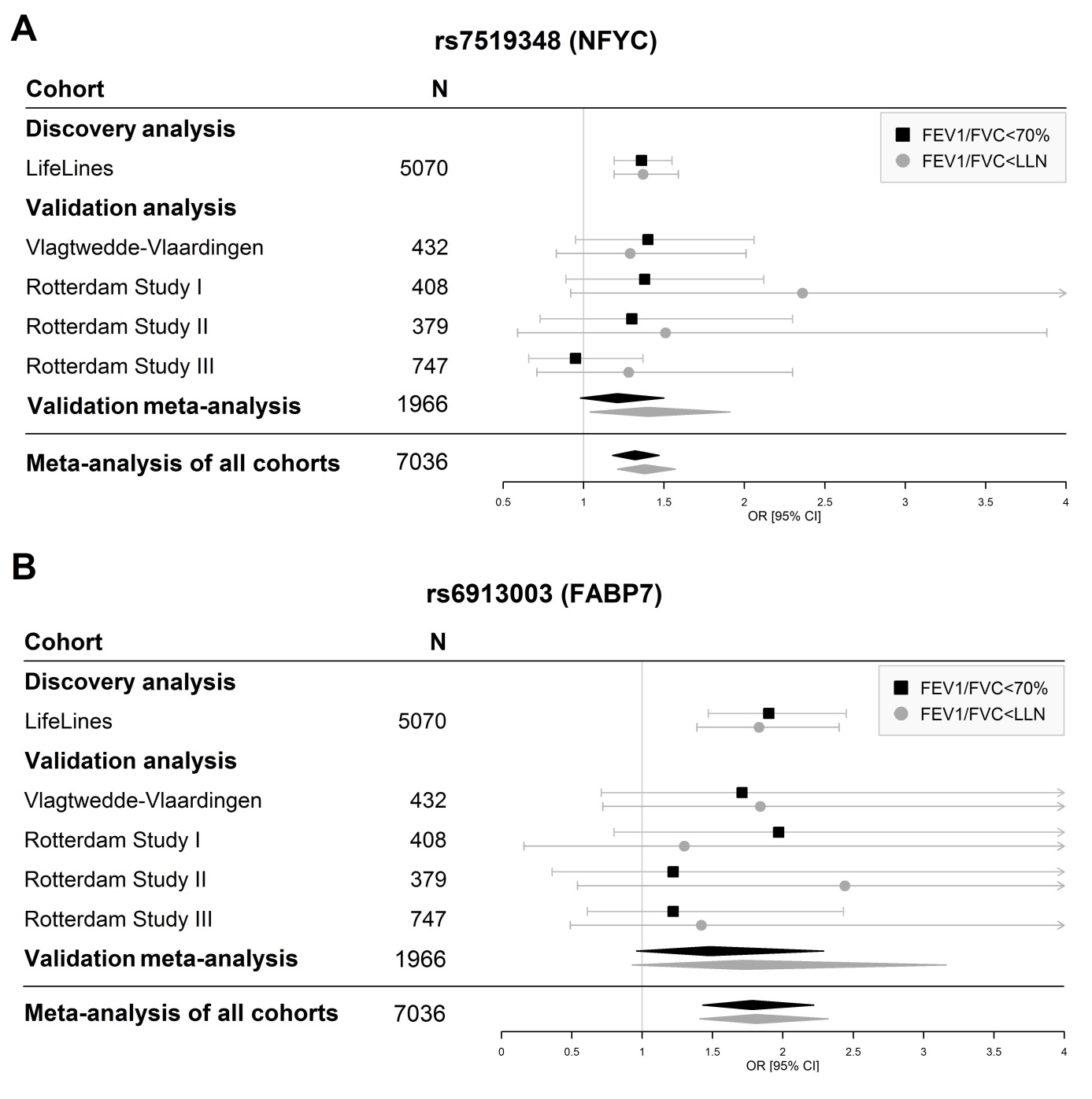


Figure S6. Forrest plots showing the associations of the two overlapping SNPs in never-smokers with both definitions of airflow obstruction. A) rs7519348 in *NFYC* and B) rs6913003 in *FABP7*.
